# Secretary bird optimization algorithm based on quantum computing and multiple strategies improvement for KELM diabetes classification

**DOI:** 10.1038/s41598-025-87285-0

**Published:** 2025-01-30

**Authors:** Yu Zhu, Mingxu Zhang, Qinchuan Huang, Xianbo Wu, Li Wan, Ju Huang

**Affiliations:** 1 School of Sports Medicine and Health, Chengdu Sport University, Chengdu, 610041 China; 2 Hospital of Chengdu University of Traditional Chinese Medicine, Chengdu, 620010 China

**Keywords:** Kernel extreme learning machine, Secretary bird optimization algorithm, Parameter optimization, Diabetes classification prediction, Quantum computing, Biological techniques, Endocrinology, Risk factors

## Abstract

The classification of chronic diseases has long been a prominent research focus in the field of public health, with widespread application of machine learning algorithms. Diabetes is one of the chronic diseases with a high prevalence worldwide and is considered a disease in its own right. Given the widespread nature of this chronic condition, numerous researchers are striving to develop robust machine learning algorithms for accurate classification. This study introduces a revolutionary approach for accurately classifying diabetes, aiming to provide new methodologies. An improved Secretary Bird Optimization Algorithm (QHSBOA) is proposed in combination with Kernel Extreme Learning Machine (KELM) for a diabetes classification prediction model. First, the Secretary Bird Optimization Algorithm (SBOA) is enhanced by integrating a particle swarm optimization search mechanism, dynamic boundary adjustments based on optimal individuals, and quantum computing-based t-distribution variations. The performance of QHSBOA is validated using the CEC2017 benchmark suite. Subsequently, QHSBOA is used to optimize the kernel penalty parameter $\:C$ and bandwidth $\:c$ of the KELM. Comparative experiments with other classification models are conducted on diabetes datasets. The experimental results indicate that the QHSBOA-KELM classification model outperforms other comparative models in four evaluation metrics: accuracy (ACC), Matthews correlation coefficient (MCC), sensitivity, and specificity. This approach offers an effective method for the early diagnosis and prediction of diabetes.

## Introduction

Diabetes, as an incurable chronic endocrine disease, poses a severe global challenge^1^. According to the latest data from the International Diabetes Federation (IDF), the number of diabetes patients worldwide has reached 466 million, and this number continues to grow. China, in particular, has the highest number of diabetes patients and the highest prevalence rate in the world, with a prevalence rate of 11.2%. Moreover, a significant proportion of individuals remain undiagnosed, with awareness of the condition at only 36.5%. As diabetes cannot be cured and is prone to triggering various complications, it places a substantial economic burden on individuals, families, and society. However, early detection can extend the life expectancy of patients, making classification algorithms critical in diabetes prediction^2^. Literature^3^ reported the avoidable hospitalization rates (AHs) for diabetes and further explored their relationship with primary healthcare (PHC) resources in China. The study demonstrated that PHC resources have a greater impact on AHs for diabetes with long-term complications compared to those for uncontrolled diabetes. At the same time, the potential for misclassification in diabetes prediction raises significant ethical concerns. False positive misclassifications may lead to unnecessary medical interventions, causing psychological stress, economic burdens, and wasting medical resources. False negatives, on the other hand, can delay treatment, increase the risk of complications, and affect public health prevention efforts. Such misclassification infringes on patients’ autonomy and right to informed consent, exacerbates health inequities in society, and leads to challenges in defining medical responsibility, undermining patients’ trust in the healthcare system. Therefore, there is an urgent need for technical improvements and the development of ethical guidelines to reduce misclassification risks, protect patient rights, and promote the responsible application and development of diabetes prediction technologies^4,5^.

In today’s world, the rapid development of artificial intelligence and big data technologies has led to an increasing application of machine learning in diabetes research^6–8^. Many researchers are attempting to classify diabetes using computational intelligence techniques, such as fuzzy logic and Artificial Bee Colony (ABC) algorithms^9^. Varma et al.^10^ proposed a modified decision tree based on fuzzy decision boundaries for diabetes diagnosis. Huang H proposed a self-paced learning $\:{L}_{1/2}$ logistic regression model based on absolute networks to mitigate the impact of high-noise samples in the data on model training and to provide better predictive accuracy^11^. Wei et al. employed a Mendelian randomization strategy to explore the causal relationship between type 2 diabetes and neurological diseases^12^. Polat et al.^13^ developed a cascade learning system for diabetes classification using Generalized Discriminant Analysis (GDA) and Least Squares Support Vector Machine (LS-SVM). Beloufa et al.^14^ designed a fuzzy classifier using an improved ABC optimization technique to generate fuzzy rules for diabetes datasets, thereby enhancing the accuracy of diabetes classification. These previous studies provide a theoretical foundation and prior knowledge for current research and establish important prerequisites for further investigations.

Common classifiers such as SVM and KELM have certain limitations^15^. SVM is inefficient in processing large-scale data, with high computational complexity and memory consumption in solving quadratic programming problems. It is also sensitive to data noise, which can pull the decision hyperplane, leading to overfitting. Additionally, the selection of the kernel function and parameter tuning are complex, and multi-class classification is relatively cumbersome^16^. Similarly, KELM relies on the choice of kernel function, which can impact performance if chosen poorly. Furthermore, as a neural network-based model, KELM has a complex structure and poor interpretability, making it difficult to intuitively understand the model’s decision-making process and reasoning^17^.

Kernel Extreme Learning Machine (KELM) is an extension of Extreme Learning Machine (ELM) based on kernel methods, characterized by fast training speed, good generalization ability, and suitability for complex problems^18,19^. The effectiveness of the KELM classifier is affected by two primary parameters: the kernel penalty parameter $\:\text{C}$ and the bandwidth $\:\text{c}$. Thus, it is crucial to configure these parameters correctly prior to using the classifier in real-world scenarios. Commonly, grid search techniques are employed to fine-tune the kernel penalty and bandwidth; however, this method often risks becoming stuck in local optima and can present challenges in defining suitable ranges for the parameters^20^. Optimizing the two key parameters of KELM using meta-heuristic algorithms can help quickly find suitable parameter combinations and improve the model’s classification performance^21^. For instance, Lu et al.^16^ utilized the Active Operators Particle Swarm Optimization algorithm (APSO) to obtain the optimal initial parameter set for KELM, creating an optimal KELM classifier named APSO-KELM. Wang et al.^22^ developed a novel parameter tuning approach for KELM utilizing Grey Wolf Optimization (GWO) specifically for bankruptcy prediction models. A. Yaqoob introduced a novel hybrid gene selection method that combines Harris Hawk Optimization (HHO) and Whale Optimization (WO) algorithms with deep learning to enhance feature selection and classification accuracy^23^. Abrar Yaqoob also proposed a new feature selection method that integrates Random Drift Optimization (RDO) with XGBoost, specifically designed to improve the performance of cancer classification tasks^24^. Sana Afreen introduced a new hybrid feature selection (FS) algorithm, called the Game Kernel SHapley Additive Explanation (kSHAP), which combines with Binary Social Ski Driver (bSSD), Adaptive Beta Hill Climbing (ABHC), and Late Acceptance Hill Climbing (LAHC) algorithms^25^. Abrar Yaqoob presented a new approach for gene selection and breast tumor classification using Salp Swarm Optimization (SSO) and Support Vector Machine (SVM)^26^. R. M. Aziz proposed a new Particle Swarm-Cuckoo Search (PS-CS) optimization algorithm, which leverages the strengths of Particle Swarm Optimization and Cuckoo Search algorithms to enhance network parameter optimization and train deep neural networks for crime prediction^27^. Joshi introduced a wrapper algorithm based on the Spider Monkey Optimization (SMO) algorithm combined with Genetic Algorithm (GA) to identify gene sets that improve the classification accuracy of Naive Bayes (NB) and Support Vector Machine (SVM) classifiers^28^. Li et al.^29^ presented a biogeography-based optimization technique (BBO-KELM) aimed at short-term wind power forecasting. Hu et al.^17^ optimized the regularization coefficient $\:\text{C}$ and the kernel function parameter ccc of KELM through GSWOA, employing three strategies to create a GSWOA-KELM fault diagnosis model, thus mitigating the low diagnostic accuracy that arises from manual parameter selection. Quan^30^ introduced a fault diagnosis technique that integrates Kernel Principal Component Analysis (KPCA) with an enhanced Sparrow Search Algorithm (ISSA) to optimize KELM. Han^15^ proposed a DGA method for diagnosing faults in power transformers, leveraging Harris Hawks Optimization (HHO) to enhance KELM performance. To improve the accuracy of thermal error prediction models for electrical spindles, Dai^31^ suggested a KELM thermal error modeling approach based on Snake Optimization. Han^32^ addressed issues of high randomness in power load and low prediction accuracy by proposing a short-term power load forecasting (STPLF) model optimized by an Improved Whale Optimization Algorithm (IWOA) for KELM. Yue^33^ introduced a multi-label data classification method using KELM optimized by Particle Swarm Optimization (PSO) and Butterfly Optimization Algorithm (BOA)^34^, which fully considers the optimization of model generalization capability and the number of hidden layer nodes. This approach can maintain the current time complexity of the algorithm while avoiding extensive repetitive calculations, effectively training multiple KELM hidden layer networks.

However, metaheuristic algorithms also have inevitable limitations. In 1997, David H. Wolpert and William G. Macready^35^ introduced the No Free Lunch (NFL) Theorem, which logically demonstrated that no metaheuristic algorithm can universally solve all optimization problems in the best possible way^36^. This prompted many researchers to develop new methods to address these challenges. For instance, El-Sayed M. El-Kenawy and colleagues, inspired by the behavior of Greylag Geese, proposed the Greylag Goose Optimization^37^. H. Zamani, drawing on the exceptional navigational accuracy of migratory birds during long-distance flights, introduced the Quantum Avian Navigation Algorithm (QANA)^38^. Mehta et al. introduced a multi-objective version of the Brown Bear Optimization (BBO) algorithm, which draws inspiration from the foraging behavior of brown bears, offering valuable insights into the field of multi-objective algorithms^39^. Ghanshyam G proposed a 2-archive multi-objective cuckoo search algorithm through a dual-archive strategy^40^. The Whale Optimization Algorithm (WOA)^41^, inspired by the group hunting behavior of humpback whales, incorporates three phases: search for food, contraction and encirclement, and spiral updating. Additionally, El-Kenawy and colleagues proposed the Football Optimization Algorithm (FbOA)^42^, inspired by the dynamic strategies of football teams, and Faramarzi introduced the Marine Predator Algorithm (MPA)^43^, inspired by the foraging behaviors of marine predators.

Secretary Bird Optimization Algorithm (SBOA)^44^ is a recently proposed meta-heuristic algorithm characterized by its simple structure and fast convergence. However, it faces challenges in complex problems, including imbalances between exploration and exploitation, slow convergence rates, susceptibility to local optima, and low convergence accuracy. To address these issues, this paper proposes an improved Secretary Bird Optimization Algorithm (QHSBOA) based on the PSO search mechanism, dynamic boundary adjustment based on optimal individuals, and quantum computing-based t-distribution variation. Additionally, a new diabetes diagnosis method, QHSBOA-KELM, is developed utilizing QHSBOA for classifying diabetes based on feature vectors. Table 1 summarizes the key findings of this study and compares them with recent related research. The table highlights the unique contributions of our approach, including the integration of quantum computing and multi-strategy improvements into SBOA, as well as the superior performance of the QHSBOA-KELM model in diabetes classification.

**Table 1:** Some recent research.

Method	Author	Accuracy (%)	References
FSDT	B. Chandra et al.	72.21%	^45^
GFDT	B. Chandra et al.	74.94%	^46^
RBF	Kayaer. Yildirim et al.	68.23%	^47^
GNG	Deng. Kasabov et al.	74.60%	^48^
k-NN	Ster. Dobnikar et al.	71.90	^49^
LS-SVM	Polat, Kemal et al.	78.21%	^13^
ABC-FRBC	Fayssal Beloufa et al.	76.98%	^50^
IGGF	Kamadi V.S.R.P. Varma et al.	75.8%	^4^
CS-BGWO-SVM	C. Mallika et al.	78.2%	^51^

The novelty of this study lies in the integration of quantum computing and multi-strategy enhancements into the Secretary Bird Optimization Algorithm (SBOA), resulting in an improved algorithm termed Quantum-inspired Hybrid Secretary Bird Optimization Algorithm (QHSBOA). This advancement addresses the limitations of traditional SBOA, such as slow convergence and susceptibility to local optima, which have not been explored in previous research. This approach not only enhances the optimization performance of SBOA but also provides a novel methodology for diabetes classification using Kernel Extreme Learning Machine (KELM). Compared to existing methods, our proposed QHSBOA-KELM model demonstrates superior performance in terms of accuracy, convergence speed, and stability, as evidenced by the experimental results.

The effectiveness of the proposed method is validated using the Pima Indians Diabetes Dataset (PIDD), and the results are compared with those obtained from other algorithms optimized KELM methods to further confirm the validity of the proposed diabetes diagnosis approach. The main contributions of this paper are as follows:

1. An improved QHSBOA is proposed by integrating the PSO search mechanism, dynamic boundary adjustment based on optimal individuals, and quantum computing-based t-distribution variation into SBOA.
2. The optimization performance of QHSBOA is validated through comparisons with eight advanced algorithms using the CEC2017 benchmark suite.
3. A diabetes diagnosis method, QHSBOA-KELM, is proposed based on QHSBOA, and its superiority is demonstrated through comparisons with other models.

The organization of the remaining sections of this paper is as follows: Sect. 2 introduces the principles of SBOA and the improved QHSBOA; Sect. 3 briefly validates the effectiveness of the proposed QHSBOA method using the CEC2017 benchmark suite; Sect. 4 develops the QHSBOA-KELM diabetes diagnosis method and conducts comparative validation; Sect. 5 summarizes the research findings and outlines future wo.

## Secretary bird optimization and the proposed methodology

### Secretary bird optimization algorithm (SBOA)

#### Initialization

The SBOA method is a population-based meta-heuristic approach that, like other heuristic algorithms, randomly generates a set of candidate solutions within the search space.

1 $$\:\begin{array}{c}X={\left[\begin{array}{cc}\begin{array}{ccc}{\text{x}}_{\text{1,1}}&\:{\text{x}}_{\text{1,2}}&\:\cdots\:\\\:{\text{x}}_{\text{2,1}}&\:{\text{x}}_{\text{2,2}}&\:\cdots\:\\\:\vdots&\:\vdots&\:\ddots\:\end{array}&\:\begin{array}{ccc}{\text{x}}_{1,\text{j}}&\:\cdots\:&\:{\text{x}}_{1,\text{D}}\\\:{\text{x}}_{2,\text{j}}&\:\cdots\:&\:{\text{x}}_{2,\text{D}}\\\:\vdots&\:\ddots\:&\:\vdots\end{array}\\\:\begin{array}{ccc}{\text{x}}_{\text{i},1}&\:{\text{x}}_{\text{i},2}&\:\cdots\:\\\:\vdots&\:\vdots&\:\ddots\:\\\:{\text{x}}_{\text{N},1}&\:{\text{x}}_{\text{N},2}&\:\cdots\:\end{array}&\:\begin{array}{ccc}{\text{x}}_{\text{i},\text{j}}&\:\cdots\:&\:{\text{x}}_{\text{i},\text{D}}\\\:\vdots&\:\ddots\:&\:\vdots\\\:{\text{x}}_{\text{N},\text{j}}&\:\cdots\:&\:{\text{x}}_{\text{N},\text{D}}\end{array}\end{array}\right]}_{\text{N}\times\:\text{D}\:}\end{array}$$

In this context, $\:\text{X}$ represents the population of secretary birds, $\:{\text{X}}_{\text{i}}$ denotes the position of the $\:{\text{i}}^{\text{t}\text{h}}$ secretary bird, $\:{\text{x}}_{\text{i},\text{j}}$ indicates the position information of the $\:{\text{j}}^{\text{t}\text{h}}$ problem variable for the $\:{\text{i}}^{\text{t}\text{h}}$ secretary birds,$\:\text{N}$ denotes the population size, and $\:\text{D}$ represents the dimensionality of the problem variables.

The initial positions of the Secretary Birds are randomly determined based on Eq. (2):

2 $$\:\begin{array}{c}{\text{x}}_{\text{i},\text{j}}=\left({\text{u}\text{b}}_{\text{j}}-{\text{l}\text{b}}_{\text{j}}\right)\times\:{\text{r}}_{1}+{\text{l}\text{b}}_{\text{j}}\end{array}$$

Here, $\:{\text{x}}_{\text{i},\text{j}}$ represents the initial value of the $\:{\text{j}}^{\text{t}\text{h}}$ decision variable for the $\:{\text{i}}^{\text{t}\text{h}}$ candidate solution; $\:{\text{u}\text{b}}_{\text{j}}$ and $\:{\text{l}\text{b}}_{\text{j}}$ are the maximum and minimum boundaries, respectively; and $\:{\text{r}}_{1}$ is a random number in the range (0, 1).

#### Hunting behavior (exploration)

The hunting behavior of secretary birds typically consists of three stages: prey searching $\:\left({\text{P}}_{1}\right)$,prey exhausting $\:\left({\text{P}}_{2}\right)$, and prey attacking $\:\left({\text{P}}_{3}\right)$. During the prey searching stage, Secretary Birds look for potential prey. Once prey is identified, they enter the prey exhausting stage, where they expend the prey’s stamina. With keen judgment of the prey’s movements, the Secretary Birds leisurely hover, leap, and provoke near the snake, wearing down the opponent’s energy. Once the prey’s stamina is sufficiently depleted, they initiate the attack. This process is modeled using Eqs. (3) and (4)^44^.

3 $$\:\begin{array}{l}{\text{x}}_{\text{i},\text{j}}^{\text{n}\text{e}\text{w}1}=\left\{\begin{array}{ll}{\text{P}}_{1}:{\:\text{x}}_{\text{i},\text{j}}+{\text{r}}_{2}\times\:{(\text{x}}_{{\text{r}}_{1}}-{\text{x}}_{{\text{r}}_{2}}), & if\:iter< \frac{1}{3}T\\\:{\text{P}}_{2}:\:{\text{x}}_{\text{b}\text{e}\text{s}\text{t}}+exp\left({\left(\frac{\text{i}\text{t}\text{e}\text{r}}{\text{T}}\right)}^{4}\right)\times\:\left(\text{R}\text{B}-0.5\right)\times\:\left({\text{x}}_{\text{b}\text{e}\text{s}\text{t}}-{\text{x}}_{\text{i},\text{j}}\right), & if\:\frac{1}{3}T< iter <\frac{2}{3}T\\\:{\text{P}}_{3}:{\:\text{x}}_{\text{b}\text{e}\text{s}\text{t}}+{\left(1-\frac{\text{i}\text{t}\text{e}\text{r}}{\text{T}}\right)}^{\left(2\times\:\frac{\text{i}\text{t}\text{e}\text{r}}{\text{T}}\right)}\times\:{\text{x}}_{\text{i},\text{j}}\times\:RL, & else\end{array}\right.\end{array}$$

4 $$\:\begin{array}{c}{\text{X}}_{\text{i}}=\left\{\begin{array}{ll}{\:\text{X}}_{\text{i}}^{\text{n}\text{e}\text{w}1},& if\:\:{\text{F}}_{\text{i}}^{\text{n}\text{e}\text{w}1}<{\text{F}}_{\text{i}}\\\:{\text{X}}_{\text{i}\:}, & else\end{array}\right.\end{array}$$

Here,$\:\text{i}\text{t}\text{e}\text{r}$ denotes the current iteration number, $\:\text{T}$ represents the maximum number of iterations,$\:{\:\text{X}}_{\text{i}}^{\text{n}\text{e}\text{w}1}$ indicates the new state of the $\:{\text{i}}^{\text{t}\text{h}}$ secretary bird in the first stage, $\:{\text{x}}_{\text{r}1}$ and $\:{\text{x}}_{\text{r}2}$ are random candidate solutions for the first stage iteration.$\:{\text{r}}_{2}$ is a randomly generated array of dimensions $\:1\times\:\text{D}$ from the interval [0, 1]. $\:{\text{x}}_{\text{i},\text{j}}^{\text{n}\text{e}\text{w}1}$ represents the position information of its $\:{\text{j}}^{\text{t}\text{h}}$ dimension, while $\:{\text{F}}_{\text{i}}^{\text{n}\text{e}\text{w}1}$ signifies its objective function fitness value.$\:\text{R}\text{B}$ is an array of dimensions $\:1\times\:\text{D}$ generated randomly from a standard normal distribution (mean = 0, standard deviation = 1), and $\:{\text{x}}_{\text{b}\text{e}\text{s}\text{t}}$ represents the best solution obtained so far.$\:\text{R}\text{L}$ refers to the Lévy flight function, calculated using Eq. (5).

5 $$\:\begin{array}{c}\left\{\begin{array}{c}RL=0.5\times\:Levy\left(\text{D}\text{i}\text{m}\right)\\\:Levy\left(\text{D}\text{i}\text{m}\right)=0.01\times\:\frac{\text{u}\times\:{\upsigma\:}}{|\text{v}{|}^{\frac{1}{{\upeta\:}}}}\\\:\sigma\:={\left(\frac{{\Gamma\:}\left(1+{\upeta\:}\right)\times\:\text{sin}\left(\frac{{\uppi\:}\times\:{\upeta\:}}{2}\right)}{{\Gamma\:}\left(\frac{1+{\upeta\:}}{2}\right)\times\:{\upeta\:}\times\:2\left(\frac{{\upeta\:}-1}{2}\right)}\right)}^{\frac{1}{{\upeta\:}}}\end{array}\right.\end{array}$$

In this equation, $\:{\upeta\:}$ is a fixed constant of 1.5. The variables $\:\text{u}$ and $\:\text{v}$ are random numbers within the interval [0, 1], and $\:{\Gamma\:}$ represents the gamma function, with $\:{\upeta\:}$ also equal to 1.5.

#### Escape strategy (exploitation)

Secretary birds may face attacks from predators or attempts to steal their food. Being highly intelligent, they often employ evasion strategies to protect themselves or their food. These strategies are primarily divided into two types: one involves flying or running to escape$\:\left({\text{S}}_{1}\right)$,while the other involves using environmental colors or structures for camouflage$\:\left({\text{S}}_{2}\right)$, to make it harder for predators to detect them^44^. This process is modeled using Eqs. (6) and (7).

6 $$\:\begin{array}{c}{\:\text{x}}_{\text{i},\text{j}}^{\text{n}\text{e}\text{w}2}=\left\{\begin{array}{ll}{{\text{S}}_{1}:\:\:\text{x}}_{\text{b}\text{e}\text{s}\text{t}}+\left(2\times\:\text{R}\text{B}-1\right)\times\:{\left(1-\frac{\text{i}\text{t}\text{e}\text{r}}{\text{T}}\right)}^{2}\times\:{\text{x}}_{\text{i},\text{j}},&if\:\:q<{\text{r}}_{3}\\\:{\text{S}}_{2}:{\:\:\text{x}}_{\text{i},\text{j}}+{\text{r}}_{4}\times\:\left({\text{x}}_{\text{r}\text{a}\text{n}\text{d}}-\text{l}\times\:{\text{x}}_{\text{i},\text{j}}\right)\:,&else\end{array}\right.\end{array}$$

7 $$\:\begin{array}{c}{\text{X}}_{\text{i}}=\left\{\begin{array}{ll}{\:\text{X}}_{\text{i}}^{\text{n}\text{e}\text{w}2},& if\:\:{\text{F}}_{\text{i}}^{\text{n}\text{e}\text{w}2}<{\text{F}}_{\text{i}}\\\:{\text{X}}_{\text{i}\:},& else\end{array}\right.\end{array}$$

In this equation, $\:\text{q}=0.5,{\text{r}}_{3}$ and $\:{\text{r}}_{4}$ represent arrays of dimensions $\:(1\times\:\text{D})$ randomly generated from a normal distribution. $\:{\text{x}}_{\text{r}\text{a}\text{n}\text{d}}$ denotes a random candidate solution in the current iteration, and $\:\text{l}$ is a randomly chosen integer of either 1 or 2.

### Proposed secretary bird optimization algorithm

#### PSO search mechanism

In its early stages, the Secretary Bird Optimization Algorithm (SBOA) exhibits limited exploration capability and a lack of diversity within the population, which can lead to suboptimal solution quality. To enhance exploration ability, improve population diversity^52^, and increase solution quality, this study integrates Particle Swarm Optimization (PSO) by introducing the concept of velocity, providing SBOA with a new search mechanism. During early iterations, secretary bird individuals update their positions, with velocity updates introducing additional randomness. This helps prevent premature convergence of the algorithm and encourages exploration of new areas, thereby enhancing population diversity. By dynamically adjusting each individual’s velocity and position, this approach can effectively balance global exploration and local exploitation^53,54^. Conducting broader searches during the early iterations helps identify potential high-quality solutions. The calculation formula (8) is as follows:

8 $$\begin{aligned}v_{i,j}&=w\times v_{i,j}+c_1\times\alpha_1\times(x_{pbest}-x_{i,j})+c_2\times\alpha_2\times(x_{gbest}-x_{i,j})\\x_{i,j}^{\text{new}1}&=x_{i,j}+v_{i,j}\end{aligned}$$

In this context, $\:\text{w}$ represents the inertia weight, which controls the influence of the particle’s previous velocity. It is typically decreased gradually during the iteration process to enhance the balance between global and local search. In this study, $\:\text{w}=\left(1-\frac{\text{i}\text{t}\text{e}\text{r}}{\text{T}}\right).{{\upalpha\:}}_{1}$ and $\:{{\upalpha\:}}_{2}$ are random numbers between [0, 1], introduced to increase randomness and ensure diversity in the particle search. $\:{\text{c}}_{1}$ is the individual learning factor, indicating the particle’s awareness of its own best position, while $\:{\text{c}}_{2}$ is the social learning factor, reflecting the particle’s awareness of the global best position. $\:{\text{x}}_{\text{p}\text{b}\text{e}\text{s}\text{t}}$ denotes the historical best position (individual best position), and $\:{\text{x}}_{\text{g}\text{b}\text{e}\text{s}\text{t}}$ refers to the current best position in the population (global best position).

**Fig. 1:**
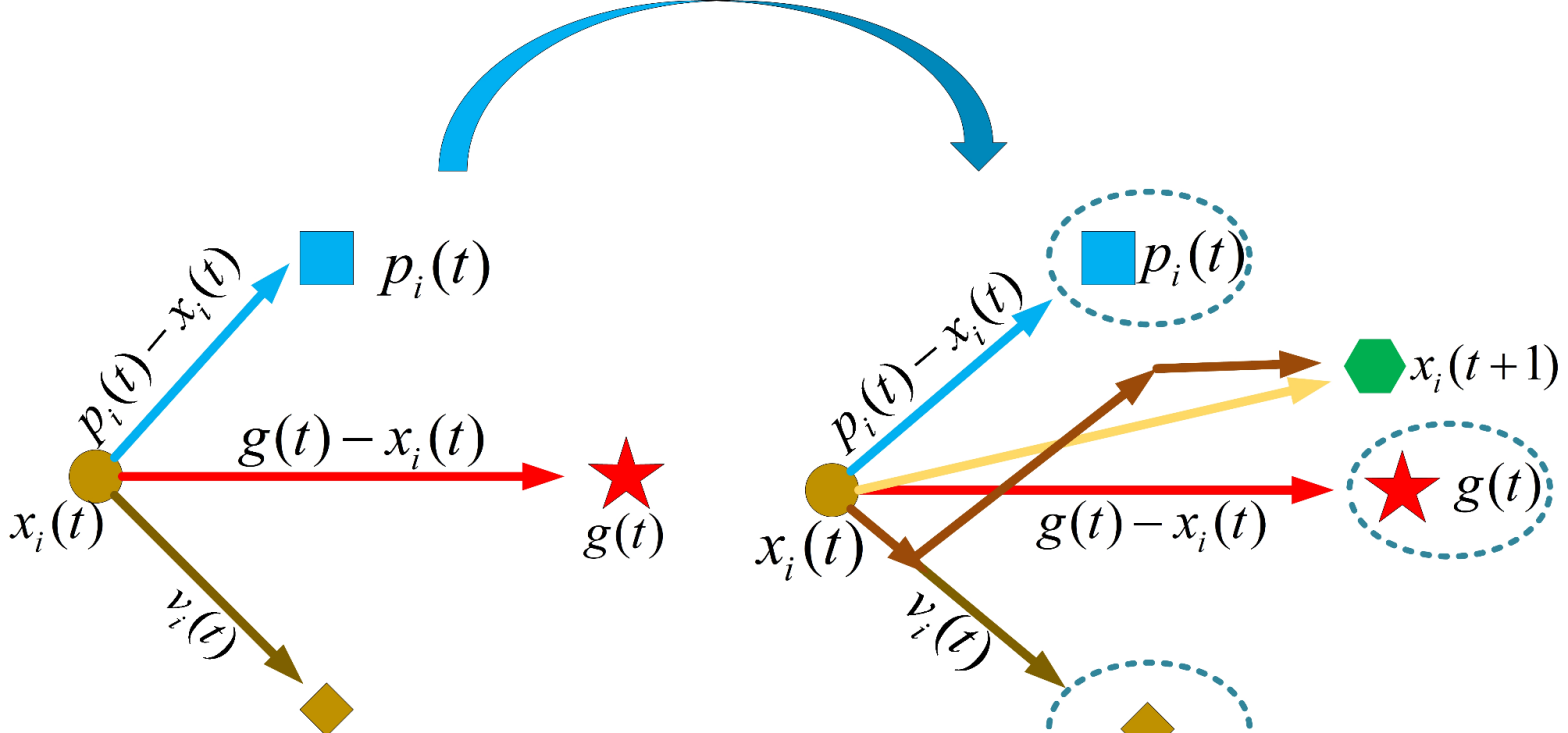
PSO search mechanism.

In this study, the PSO search mechanism was employed in the third phase of the predation strategy of SBOA, with additional randomness introduced to stimulate a broader global search. As shown in Fig. 1, this mechanism combines information from the individual best position and the global best position to facilitate the search, helping to avoid local optimization and increase population diversity. This approach not only accumulates more diverse and high-quality search experiences for SBOA but also balances global exploration and local exploitation more effectively through dynamic adjustments in search behavior.

#### Dynamic boundary adjustment based on optimal individual

In swarm intelligence algorithms, boundary control is essential as it constrains the search space, helping to avoid challenges such as ineffective searches, premature convergence, and getting trapped in local optima^55^. These issues can occur when the search process strays from feasible solutions or surpasses problem constraints. If the search space boundaries are too restrictive, it may limit the algorithm’s capacity to explore a wider solution space, increasing the risk of falling into local optima^56^. Thus, establishing suitable boundaries can improve the likelihood of finding the global optimum, minimize ineffective searches, and efficiently prevent the expenditure of time and resources on invalid solutions.

**Fig. 2:**
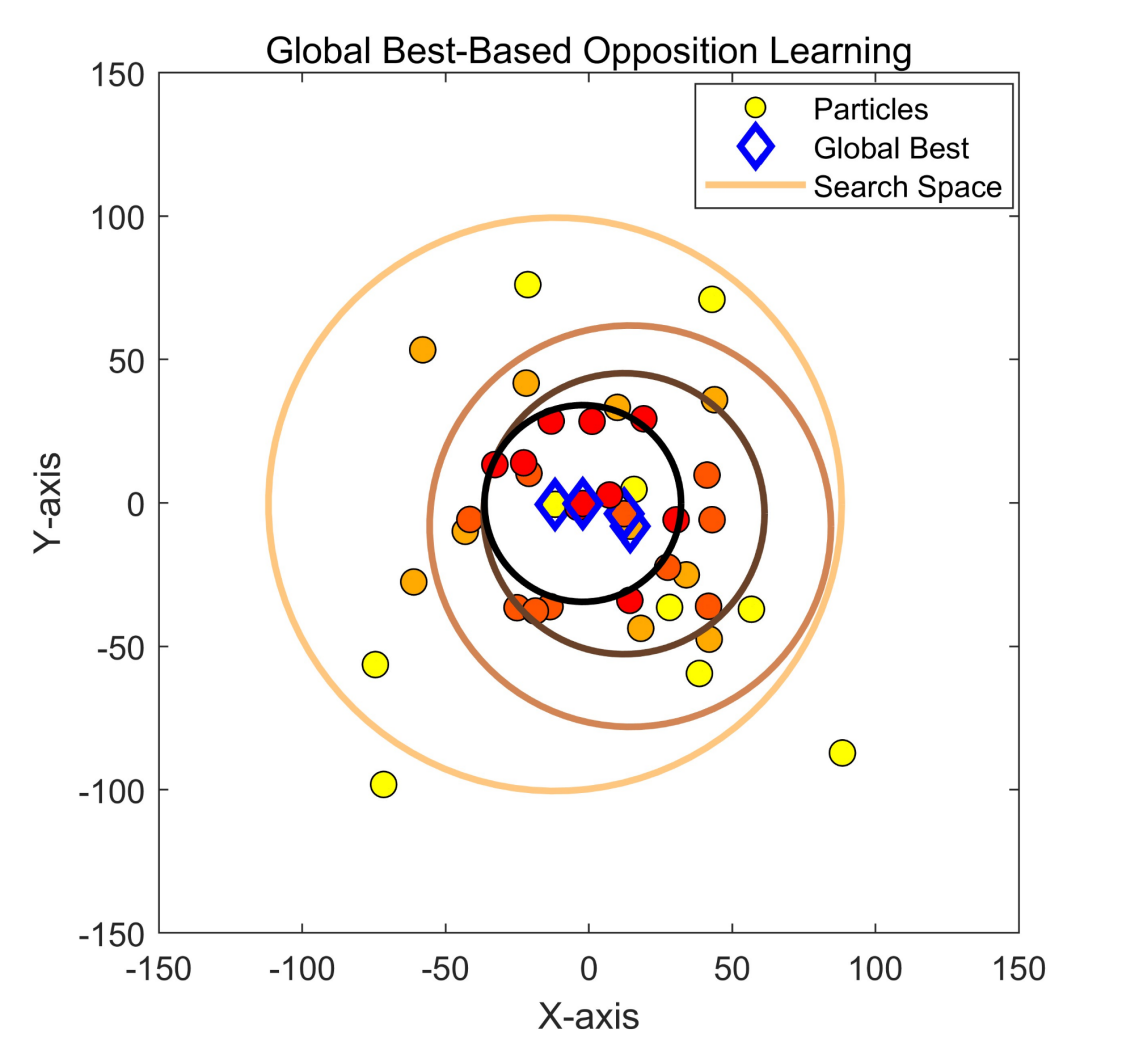
Dynamic boundary adjustment.

In certain cases, fixed boundaries may restrict the search process, preventing the algorithm from fully utilizing the information of the current optimal solution^57^. This study introduces $\:{\text{x}}_{\text{p}\text{b}\text{e}\text{s}\text{t}}$ (the best individual within the current range) into the boundary constraints, allowing for a more flexible search process. This enables the algorithm to conduct more detailed searches in areas close to the current optimal solution, thereby improving the efficiency and quality of optimization. This means that the search boundaries are influenced not only by fixed lower and upper bounds ($\:\text{l}\text{b}$ and $\:\text{u}\text{b}$) but also by the current optimal individual, as illustrated in Fig. 2. This approach allows the search process to focus more on the vicinity of known high-quality solutions, increasing the opportunity to explore even better solutions, as represented mathematically in Eq. (9).

9 $$\begin{aligned}x_{i,j}(iter + 1)& = \frac{(x_{pbest} + ub)}{2}\\x_{i,j}(iter + 1) &= \frac{(x_{pbest} + lb)}{2}\end{aligned}$$

The improved boundary constraint method enhances the flexibility and efficiency of optimization algorithms. By integrating the current optimal solution, the algorithm not only avoids the limitations of searching within fixed boundaries but also increases the chances of finding better solutions in promising regions.

#### Quantum computing based t-distribution variation

The quantum-inspired evolutionary algorithm was first proposed by Han and Kim^58^. They utilized the properties of quantum bits, which distinguish themselves from traditional computing by employing quantum bits instead of conventional binary bits. Unlike binary bits, quantum bits can exist in multiple states simultaneously, allowing for effective encoding of individuals. This approach ensures good exploration of the search space, even with a limited number of initial points. Rui Wu further optimized circular chaotic mapping using quantum bit features to initialize the population, resulting in improved initial individuals and enhanced accuracy in the algorithm’s optimization process^59,60^.

In this paper, we propose a novel mutation strategy that utilizes quantum computing interference mutation factors to perform various operations on the optimal position $\:{\text{X}}_{\text{b}\text{e}\text{s}\text{t}}$, there by enhancing the algorithm’s ability to escape local optima. Quantum bits, or qubits, are the fundamental units of information in quantum computing. They can be categorized into single qubits, double qubits, and multi-qubits; our proposed method employs single qubits. The state of a single qubit is represented by a unit two-dimensional column vector$$\:\left[\begin{array}{c}\alpha\:\\\:\beta\:\end{array}\right]$$, where $\:{{\upalpha\:}}^{2}+{{\upbeta\:}}^{2}=1$.On this basis, the quantum state vectors $$\:\left[\begin{array}{c}0\\\:1\end{array}\right]$$ and $$\:\left[\begin{array}{c}1\\\:0\end{array}\right]$$ play a special role, as any quantum state vector can be expressed as a superposition of these two states:$$\:\left|{\uppsi\:}\right|={\upalpha\:}\left[\begin{array}{c}1\\\:0\end{array}\right]+{\upbeta\:}\left[\begin{array}{c}0\\\:1\end{array}\right]$$, where $\:{\uppsi\:}$ represents the quantum bit. Thus, we can leverage the aforementioned properties of quantum mechanics to construct the mutation factor, starting by establishing a quantum space composed of multiple qubits that matches the dimensionality of the problem at hand^61^:

10 $$\:\begin{array}{c}\left|{\text{Z}}_{\text{R}}\right\rangle=\left[\begin{array}{c}{{\upalpha\:}}_{1}\\\:{{\upbeta\:}}_{1}\end{array}\:\:\begin{array}{c}{{\upalpha\:}}_{1}\cdots\:{{\upalpha\:}}_{\text{D}}\\\:{{\upbeta\:}}_{2}\cdots\:{{\upbeta\:}}_{\text{D}}\end{array}\right]\end{array}$$

Next, the optimal position $\:{\text{X}}_{\text{b}\text{e}\text{s}\text{t}}$ needs to be mapped into the quantum space. This paper accomplishes the mapping using the following method:

11 $$\begin{aligned}trnd(x,n) &= \frac{\Gamma\left(\frac{n + 1}{2}\right)}{\sqrt{n\pi}\times\Gamma\left(\frac{n}{2}\right)}\left(1+\frac{x^{2}}{n}\right)^{-\frac{n + 1}{2}}\\\Gamma(a) &= \int_{0}^{\infty}x^{a - 1}e^{-x}dx\end{aligned}$$

Here, $\:{\text{X}}_{\text{b}\text{e}\text{s}\text{t}}$ represents the value of the optimal global position, and $\:||{\text{X}}_{\text{b}\text{e}\text{s}\text{t}}||$ denotes the magnitude of $\:{\text{X}}_{\text{b}\text{e}\text{s}\text{t}}$. The probability $\:\text{P}$ is set to either 1 or -1 to ensure the diversity of the mutated points. After the transformation, the quantum space can be converted into the solution space using the following Eq. (11):

12 $$\:\begin{array}{c}\frac{{\text{X}}_{\text{t}\text{e}\text{m}\text{a}}={\upalpha\:}\bullet\:||{\text{X}}_{\text{b}\text{e}\text{s}\text{t}}||}{{\text{X}}_{\text{t}\text{e}\text{m}\text{b}}={\upbeta\:}\bullet\:||{\text{X}}_{\text{b}\text{e}\text{s}\text{t}}||}\end{array}$$

To more thoroughly explore the solution space, this paper employs quantum rotation techniques^62^ to rotate $\:\left|{\text{Z}}_{\text{R}}\right\rangle$,, thereby increasing the diversity of the population and avoiding convergence to local optima. The specifics are as follows:

13 $$\:\begin{array}{c}{\text{R}}_{{\uptheta\:}}=\left[\begin{array}{c}\text{cos}{\uptheta\:}-\text{sin}{\uptheta\:}\\\:\text{sin}{\uptheta\:}\text{cos}{\uptheta\:}\end{array}\right]\end{array}$$

14 $$\:\begin{array}{c}\left|{\text{Z}}_{\text{R}}\right\rangle={\text{R}}_{{\uptheta\:}}\left[\begin{array}{c}{{\upalpha\:}}_{1}\\\:{{\upbeta\:}}_{1}\end{array}\:\:\begin{array}{c}{{\upalpha\:}}_{1}\cdots\:{{\upalpha\:}}_{\text{D}}\\\:{{\upbeta\:}}_{2}\cdots\:{{\upbeta\:}}_{\text{D}}\end{array}\right]\end{array}$$

Where $\:{\uptheta\:}\:\in\:\:[0,\:2{\uppi\:}]$. Then, the mutation operation is performed using the following Eq. (14):

15 $$\:\begin{array}{c}{\text{X}}_{\text{b}\text{e}\text{s}\text{t}}\left\{\begin{array}{c}{\text{X}}_{\text{b}\text{e}\text{s}\text{t}}+trnd\left(\text{i}\text{t}\text{e}\text{r}\right)\times\:{\text{X}}_{{\upalpha\:}}\\\:{\text{X}}_{\text{b}\text{e}\text{s}\text{t}}+trnd\left(\text{i}\text{t}\text{e}\text{r}\right)\times\:{\text{X}}_{{\upbeta\:}}\\\:{\text{X}}_{\text{b}\text{e}\text{s}\text{t}}+trnd\left(\text{i}\text{t}\text{e}\text{r}\right)\times\:{\text{X}}_{\text{R}{\upalpha\:}}\\\:{\text{X}}_{\text{b}\text{e}\text{s}\text{t}}+trnd\left(\text{i}\text{t}\text{e}\text{r}\right)\times\:{\text{X}}_{\text{R}{\upbeta\:}}\end{array}\right.\end{array}$$

Here, $\:{\text{X}}_{\text{R}{\upalpha\:}}$ and $\:{\text{X}}_{\text{R}{\upbeta\:}}$ represent the points mapped to the solution space of the qubits after rotation, and $\:\text{t}\text{r}\text{n}\text{d}\left(\text{i}\text{t}\text{e}\text{r}\right)$ is the variance factor following a $\:\text{t}$-distribution with degrees of freedom equal to the current iteration number $\:\text{i}\text{t}\text{e}\text{r}$. Individual positions are updated through an elite selection process.

**Fig. 3:**
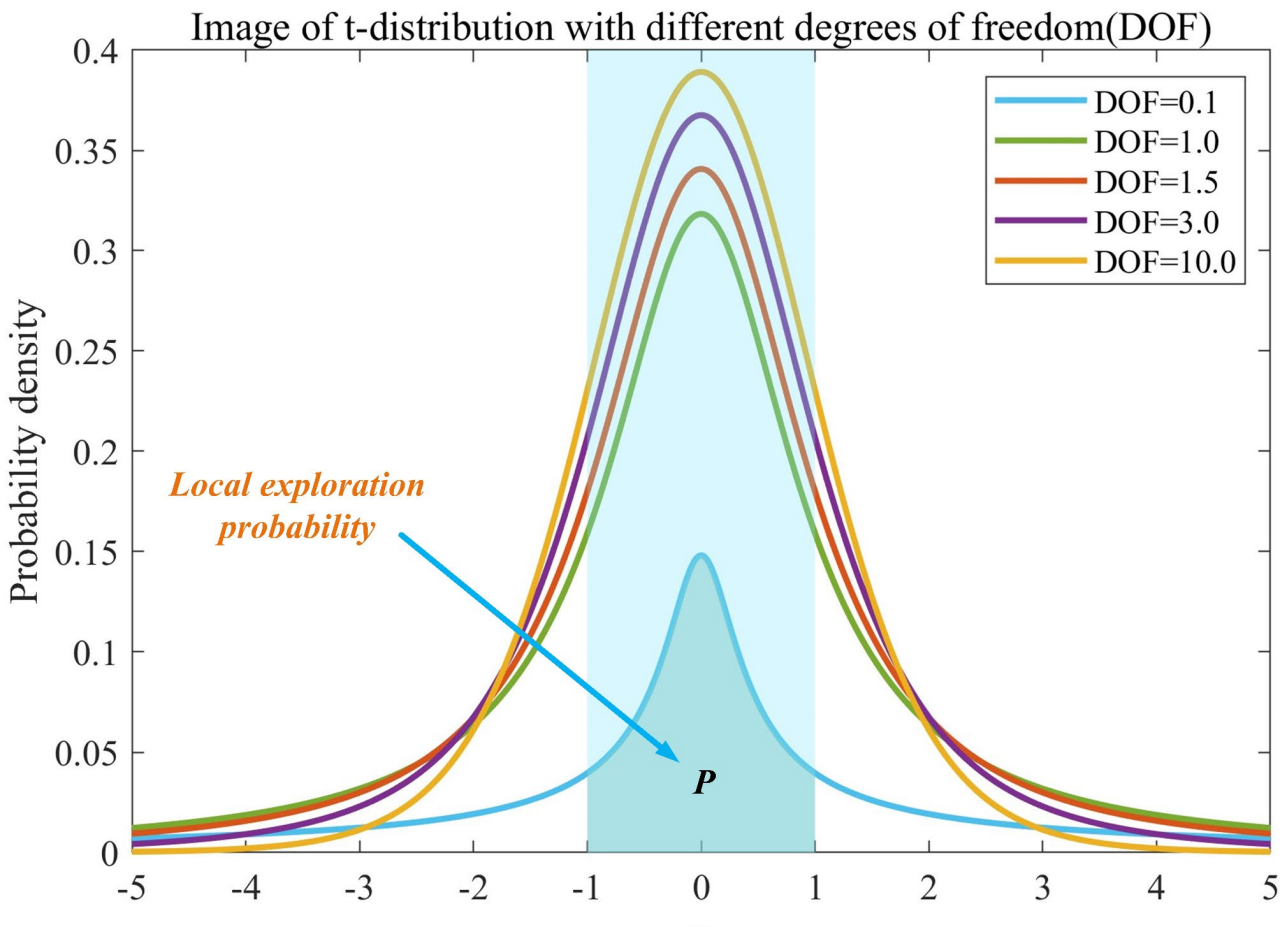
Different degrees of freedom $\:\text{t}$ distribution.

The $\:\text{t}$-distribution^63^, often referred to as Student’s t-distribution, is a widely used sampling distribution in statistics. Its shape is influenced by the degrees of freedom: as the degrees of freedom decrease, the curve flattens. When the degrees of freedom are set to 1, the $\:\text{t}$-distribution resembles a Gaussian distribution; conversely, as the degrees of freedom increase toward infinity, it approaches the standard normal distribution. The probability density function is represented in Eq. (15). Figure 3 illustrates the distribution under different degrees of freedom. In the mutation operation of the algorithm, when the mutation factor approaches 0, the algorithm tends to favor local exploitation; conversely, when the mutation factor is larger, it emphasizes global exploration. For ease of observation, we define the mutation factor to follow a $\:\text{t}$-distribution within the range [− 1, 1] to enhance the algorithm’s focus on local exploitation. When the mutation factor lies in the ranges $\:\left[-{\infty\:},-1\right]\bigcup\:\left[1,+{\infty\:}\right]$ the algorithm shifts its focus towards global exploration^64^.

16 $$\begin{aligned}trnd(x,n) &= \frac{\Gamma\left(\frac{n + 1}{2}\right)}{\sqrt{n\pi}\times\Gamma\left(\frac{n}{2}\right)}\left(1+\frac{x^{2}}{n}\right)^{-\frac{n + 1}{2}}\\\Gamma(a) &= \int_{0}^{\infty}x^{a - 1}e^{-x}dx\end{aligned}$$

As shown in Fig. 3, when the degrees of freedom of the $\:\text{t}$-distribution are 0.1, the values of the mutation factor guide the algorithm towards local exploration with a probability $\:\text{P}\:=\:{\int\:}_{-1}^{1}\text{t}\text{r}\text{n}\text{d}\left(\text{i}\text{t}\text{e}\text{r},\text{x}\right)\text{d}\text{x}$. This indicates that the area between $\:\left(\text{x}=-1\right)$ and $\:\left(\text{x}=1\right)$, bounded by the $\:\text{t}$-distribution and the x-axis, determines this probability, while $\:\text{t}\text{r}\text{n}\text{d}\left(\text{i}\text{t}\text{e}\text{r}\right)$ represents the probability density function of the $\:\text{t}$-distribution with degrees of freedom equal to $\:\text{i}\text{t}\text{e}\text{r}$. The probability that guides the algorithm towards global search is $\:\left(1-\text{P}\right)$.

From Fig. 3,it can be observed that as the degrees of freedom increase, the probability $\:\text{P}$ of guiding the algorithm towards local exploration also rises significantly. This paper aims to provide a larger search space for the algorithm during the initial iteration phase, while more aggressively pursuing local exploration in later stages to enhance iteration speed and ensure convergence. Therefore, the design in this paper determines the value of the degrees of freedom based on the number of iterations.

The pseudo code for Quantum computing based t-distribution variation is shown in Algorithm 1.

**Algorithm 1:**
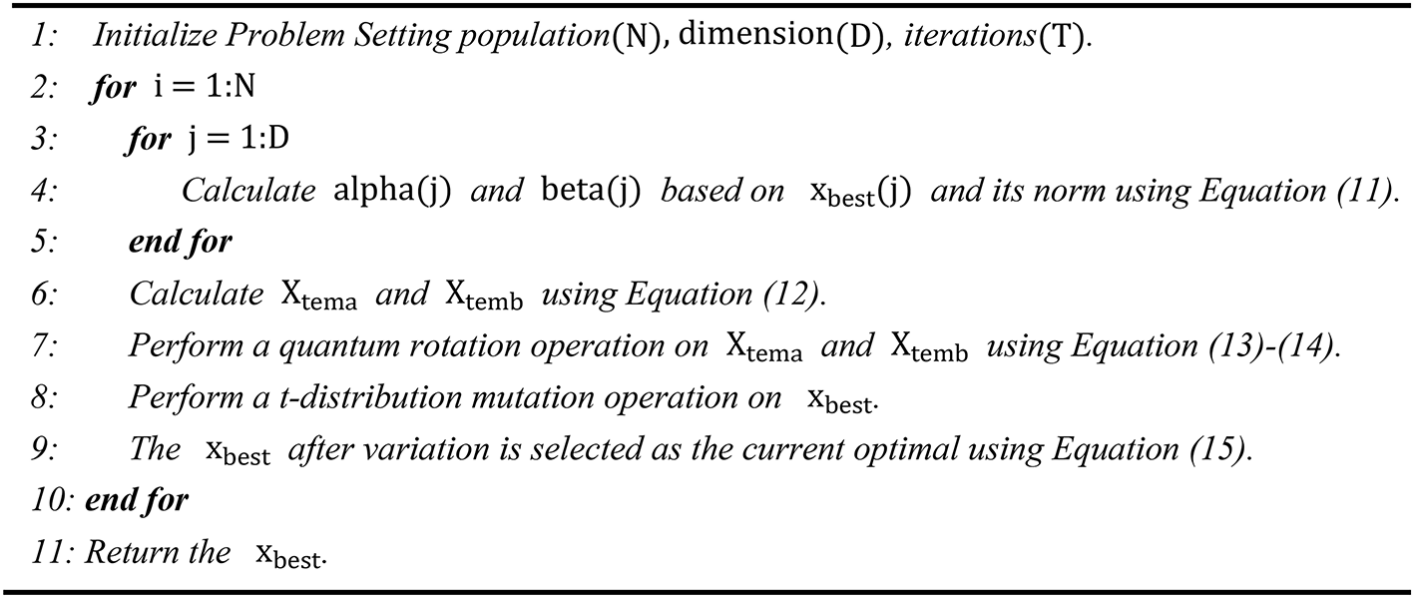
Pseudo-code of quantum computing based t-distribution variation.

In summary, the position updates for the hunting phase and the escaping phase of MSBOA are carried out using Eq. (16) and Eq. (6), respectively.

17 $$\:\begin{array}{c}{\text{x}}_{\text{i},\text{j}}^{\text{n}\text{e}\text{w}1}=\left\{\begin{array}{ll}{\text{P}}_{1}:{\:\text{x}}_{\text{i},\text{j}}+{\text{r}}_{2}\times\:{(\text{x}}_{{\text{r}}_{1}}-{\text{x}}_{{\text{r}}_{2}}),&if\:iter<\frac{1}{3}T\\\:{\text{P}}_{2}:{\text{x}}_{\text{i},\text{j}}+{\text{v}}_{\text{i},\text{j}}\:,&if\:\frac{1}{3}T<iter<\frac{2}{3}T\\\:{\text{P}}_{3}:{\:\text{x}}_{\text{b}\text{e}\text{s}\text{t}}+{\left(1-\frac{\text{i}\text{t}\text{e}\text{r}}{\text{T}}\right)}^{\left(2\times\:\frac{\text{i}\text{t}\text{e}\text{r}}{\text{T}}\right)}\times\:{\text{x}}_{\text{i},\text{j}}\times\:RL& else\end{array}\right.\end{array}$$

Based on the above discussion, the pseudocode for QHSBOA is presented in Algorithm 2.

**Algorithm 2:**
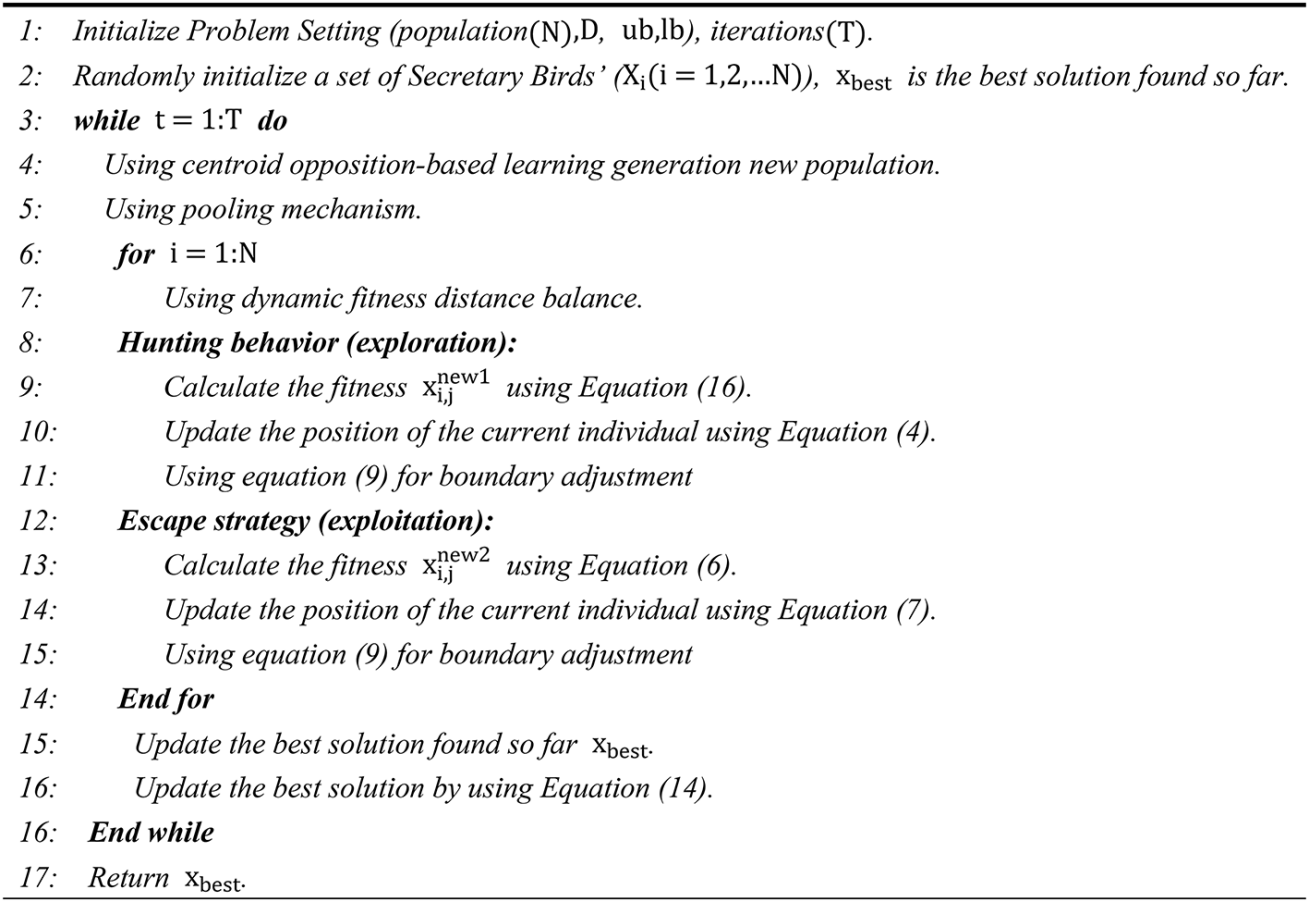
Pseudo-code of QHSBOA.

## Numerical experiments

In this subsection, the effectiveness of the proposed QHSBOA is validated through comparative experiments with other algorithms on the CEC2017 test functions^65^.

### Ablation experiment

**Fig. 4:**
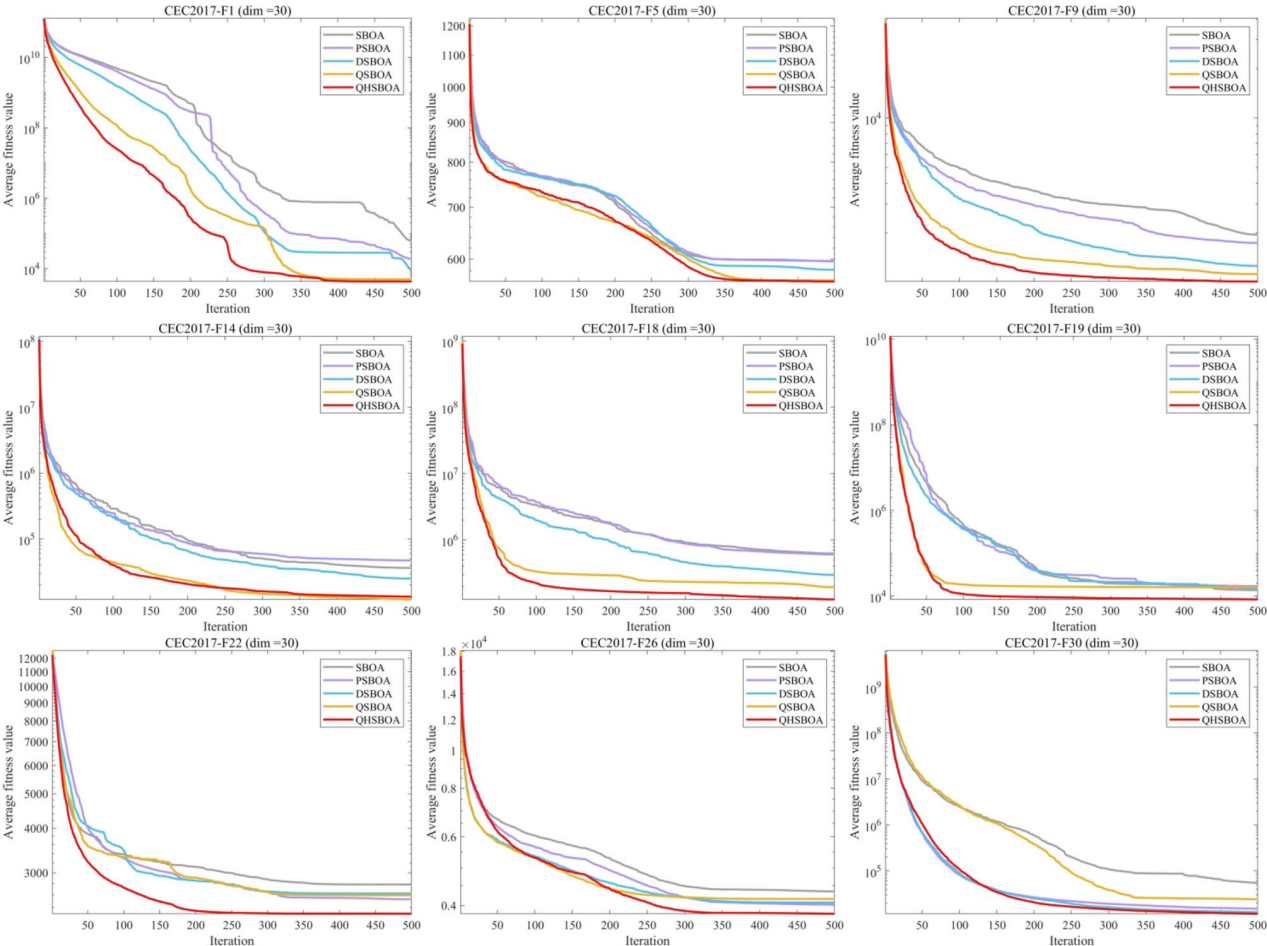
Comparison of different improvement strategies.

This section provides a detailed analysis of the impact of three proposed enhancement strategies on SBOA. These strategies include the PSO Search Mechanism, Dynamic Boundary Adjustment Based on the Optimal Individual, and Quantum Computing-Based t-Distribution Variation. Based on these strategies, three SBOA variants were introduced: PSBOA, DSBOA, and QSBOA. As shown in the experimental results in Fig. 4, each strategy improves SBOA’s convergence accuracy and speed, with the combination of all three strategies in QHSBOA demonstrating the most remarkable performance. Specifically, when dealing with unimodal and multimodal functions, PSBOA and DSBOA yield relatively consistent results, both enhancing the algorithm’s convergence speed and accuracy. Among them, QSBOA achieves the most significant improvements, greatly accelerating the algorithm’s convergence. Moreover, when handling more complex hybrid modal functions, the performance of PSBOA declines, whereas DSBOA and QSBOA exhibit enhanced effectiveness. Finally, QHSBOA, which integrates all three strategies, demonstrates superior performance across most functions.

In summary, QHSBOA successfully addresses challenges such as slow convergence speed and premature convergence. It delivers outstanding results on benchmark functions, attributed to the effectiveness of the three proposed strategies.

### Algorithm parameter settings

This section assesses the performance of the proposed QHSBOA using the CEC2017 test suite, recognized as one of the most challenging benchmarks for numerical optimization competitions, and compares it with various other algorithms. The algorithms considered for comparison include Particle Swarm Optimization (PSO)^66^, Grey Wolf Optimizer (GWO)^67^, Whale Optimization Algorithm (WOA)^41^, Dung Beetle Optimizer (DBO)^68^, Golden jackal optimization^69^, Black-winged Kite Algorithm (BKA)^70^, Crested Porcupine Optimizer (CPO)^71^, and Secretary Bird Optimization Algorithm(SBOA)^44^. The parameters for each algorithm are detailed in Table 2. To ensure experimental fairness and eliminate randomness, a fixed population size of 30 was maintained, with a maximum of 500 iterations. Each algorithm was executed independently 30 times, and the results were statistically analyzed to determine the average (Ave), standard deviation (Std), and ranking (Rank), with the top results emphasized in bold. All experiments were conducted on a computing system featuring the Windows 10 operating system, equipped with a 13th Intel(R) Core(TM) i5-13400 processor running at 2.5 GHz, 16GB of RAM, and utilizing MATLAB 2023b as the software platform.

### Experimental results and analysis of CEC2017 test suite

**Fig. 5:**
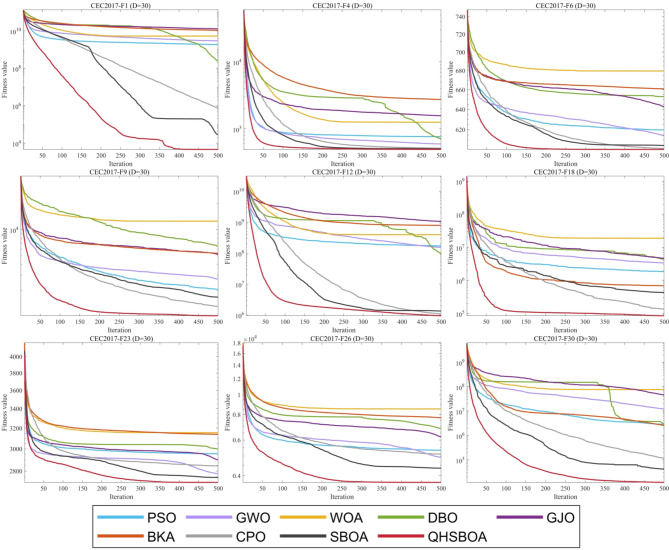
Part of the test function iterates convergence curves.

This section evaluates the performance of QHSBOA using the CEC2017 test suite. To thoroughly assess its performance, experiments were conducted in a 30-dimensional space (D = 30). The experimental statistics are presented in Table 3, which reports the average (Ave), standard deviation (Std), and ranking (Rank) for each algorithm, with the best results highlighted in bold. The convergence speed curves are illustrated in Fig. 5.

From Table 3, it is evident that QHSBOA achieved the highest number of bolded values, indicating that the proposed method is highly effective on this test suite and outperforms the majority of the comparative algorithms. Figure 5 illustrates that, regardless of whether the functions are single-modal, multi-modal, or hybrid and composite, the convergence curves of QHSBOA exhibit a consistently accelerated reduction pattern across various test functions. In contrast, algorithms such as SO, WOA, DBO, and BKA show a tendency to become trapped in local optima, failing to search for better solutions. Compared to the original SBOA and other competing optimizers, QHSBOA demonstrates a faster convergence rate, allowing it to reach solutions that are closer to the optimal one more quickly. This demonstrates that our three improvement strategies are effective, as they not only assist the algorithm in avoiding local optima but also improve its convergence speed and accuracy.

**Fig. 6:**
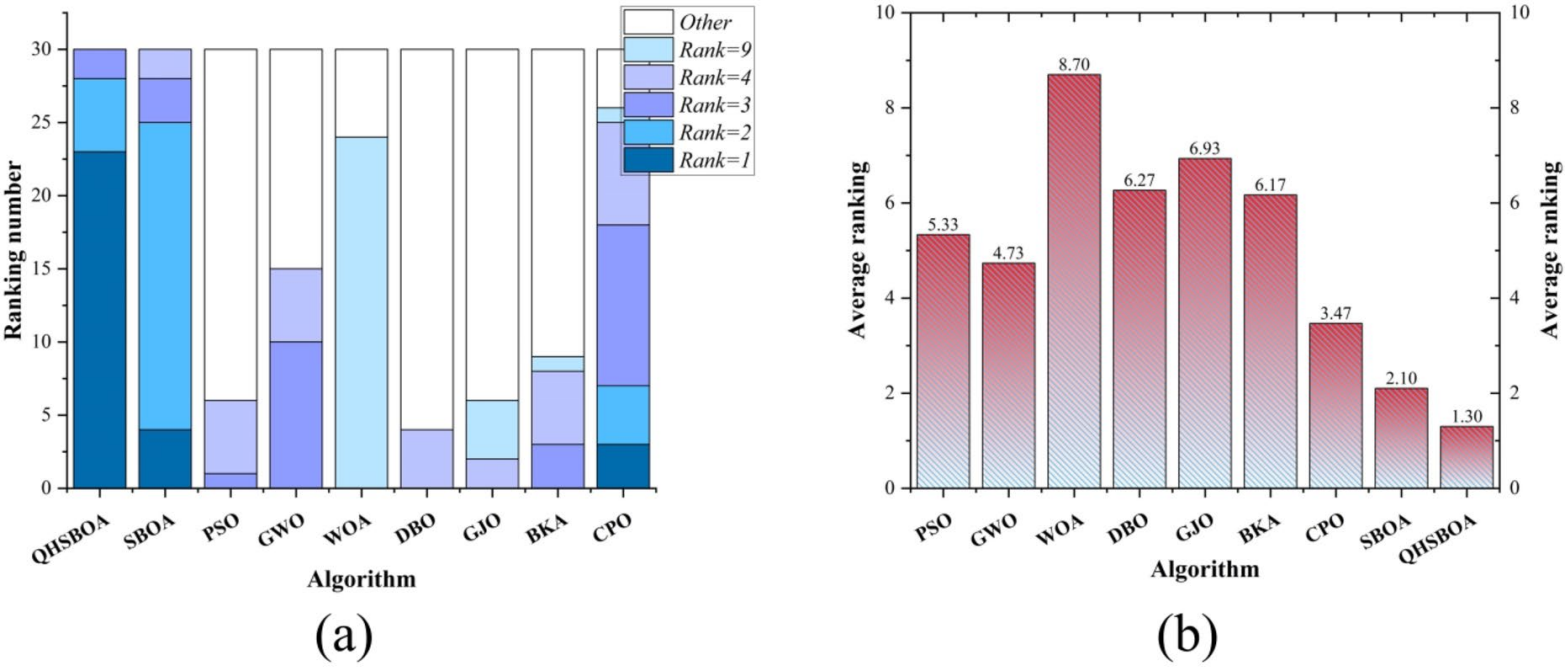
Ranking distribution and average ranking.

Additionally, to provide a clearer view of the performance of each algorithm, the ranking distribution and average values reported in Table 2 are presented in graphical form in Fig. 6. From Fig. 6a, it is evident that QHSBOA performed best on 23 functions, ranked second on 5 functions, and ranked third on 3 functions. Notably, QHSBOA maintained a position within the top three for all 30 functions, demonstrating its performance stability. Particularly when dealing with single-modal and composite functions, QHSBOA exhibited outstanding performance, while the original SBOA achieved only 4 best rankings and CPO secured just 3 best rankings, with no other algorithms attaining the best rank on any function. This indicates a significant improvement of QHSBOA over SBOA. Figure 6-(b) illustrates the average rankings of each algorithm, showing that QHSBOA achieved an average rank of 1.30, outperforming SBOA’s average rank of 2.10. WOA had the lowest average rank at 8.70, while the recently proposed CPO had an average rank of 3.47. These results collectively demonstrate the superior performance of the proposed QHSBOA.

**Table 2:** Compare algorithm parameter settings.

Algorithms	Name of the parameter	Value of the parameter
PSO	$\:{\text{c}}_{1},{\text{c}}_{2},\:\text{w}$	1.49445, 1.49445, 0.9
GWO	$\:\text{a}$	[0,2]
WOA	$\:\text{r},\:\text{l},\:\text{a}$	[0,1], [-1,1], Linear reduction from 2 to 1
DBO	$\:{\text{P}}_{\text{p}\text{e}\text{r}\text{c}\text{e}\text{n}\text{t}}$	$\:0.2$
GJO	$\:{\text{E}}_{1},{\text{E}}_{2}$	[0,1.5], [-1,1]
BKA	$\:\text{P},\:\text{r}$	0.9, [0,1]
CPO	$\:{\upalpha\:},\:{\text{N}}_{\text{m}\text{i}\text{n}},\:{\text{T}}_{\text{f}},\:\text{T}$	0.1, 80, 0.5, 2
SBOA	$\:\text{C}\text{F},\text{K},\:{\text{R}}_{1},{\text{R}}_{2}$	$\:\left[\text{0,1}\right],\left\{1,\:2\right\},\left[\text{0,1}\right],\left[\text{0,1}\right]$
QHSBOA	$\:{\text{c}}_{1},{\text{c}}_{2},\:\text{w},\text{C}\text{F},\text{K}$	$\:2,\:2,\:\left[\text{0,1}\right],\:\left[\text{0,1}\right],\left\{1,\:2\right\}$

### Wilcoxon rank sum test

To fully highlight the outstanding performance of the proposed algorithm, this section employs the Wilcoxon rank-sum test to assess whether there is a significant difference between the results of QHSBOA and other algorithms at a significance level of $\:(\text{p}\:=\:5\text{\%})$. The null hypothesis $\:\left({\text{H}}_{0}\right)$ is that there is no significant difference between the two algorithms. If $\:(\text{p}<\:5\text{\%})$, the null hypothesis is rejected, indicating a significant difference between the two algorithms; if $\:(\text{p}>\:5\text{\%})$, the null hypothesis is accepted, suggesting that the difference between the two algorithms is not significant, i.e., their performance is comparable. Table 4 presents the test results for QHSBOA and the comparison algorithms on the CEC2017 benchmark with 30 dimensions. To highlight the comparison, data with a difference greater than 5% will be bolded for emphasis.

**Table 3:** Experimental results on CEC2017 (D = 30).

F~	Metric	PSO	GWO	WOA	DBO	GJO	BKA	CPO	SBOA	QHSBOA
F1	Ave	1.8506E + 09	2.9533E + 09	5.2647E + 09	2.3650E + 08	1.2916E + 10	1.0654E + 10	7.4969E + 05	2.8391E + 04	**4.6213E + 03**
	Std	1.2401E + 09	1.8114E + 09	1.8709E + 09	2.1434E + 08	5.2594E + 09	8.7860E + 09	5.7737E + 05	2.2337E + 04	**5.2046E + 03**
	Rank	5	6	7	4	9	8	3	2	**1**
F2	Ave	5.1647E + 34	8.6677E + 32	2.1849E + 36	9.8895E + 33	9.7433E + 34	1.8383E + 39	9.8941E + 19	2.4656E + 17	**8.0595E + 12**
	Std	2.8270E + 35	3.8269E + 33	9.1663E + 36	3.6767E + 34	3.6706E + 35	9.1119E + 39	3.7874E + 20	5.4853E + 17	**1.7171E + 13**
	Rank	4	6	9	7	8	5	3	2	**1**
F3	Ave	6.8101E + 04	6.6972E + 04	2.9075E + 05	9.0539E + 04	6.1890E + 04	3.7932E + 04	6.6631E + 04	2.7888E + 04	**3.7551E + 03**
	Std	2.2316E + 04	1.2658E + 04	6.8479E + 04	1.8944E + 04	8.2527E + 03	1.8260E + 04	1.6132E + 04	8.1671E + 03	**2.1079E + 03**
	Rank	6	5	9	8	4	3	7	2	**1**
F4	Ave	7.6449E + 02	5.9528E + 02	1.2583E + 03	7.0752E + 02	1.5712E + 03	2.7448E + 03	5.1526E + 02	5.0407E + 02	**4.9346E + 02**
	Std	3.8696E + 02	5.9822E + 01	3.3416E + 02	1.6800E + 02	8.5630E + 02	3.7221E + 03	**2.0814E + 01**	2.2995E + 01	3.2019E + 01
	Rank	6	4	8	5	9	7	3	2	**1**
F5	Ave	7.2201E + 02	6.1847E + 02	8.6628E + 02	7.5140E + 02	7.2587E + 02	7.5904E + 02	6.8924E + 02	6.0443E + 02	**5.5592E + 02**
	Std	2.8951E + 01	2.5375E + 01	5.9781E + 01	5.7592E + 01	4.5817E + 01	5.1276E + 01	1.6153E + 01	3.7040E + 01	**1.5220E + 01**
	Rank	6	3	9	7	5	8	4	2	**1**
F6	Ave	6.1980E + 02	6.1406E + 02	6.7966E + 02	6.5292E + 02	6.4251E + 02	6.6091E + 02	6.0194E + 02	6.0483E + 02	**6.0112E + 02**
	Std	5.9636E + 00	4.6893E + 00	1.2734E + 01	1.1667E + 01	1.0979E + 01	8.1120E + 00	**9.4808E-01**	3.3736E + 00	1.3564E + 00
	Rank	5	4	9	7	6	8	2	3	**1**
F7	Ave	9.9001E + 02	9.0696E + 02	1.3356E + 03	1.0140E + 03	1.0720E + 03	1.2378E + 03	9.4340E + 02	8.9504E + 02	**7.9539E + 02**
	Std	3.0657E + 01	4.8683E + 01	9.7435E + 01	9.8207E + 01	7.7352E + 01	7.9811E + 01	**1.8618E + 01**	5.0721E + 01	2.2835E + 01
	Rank	5	3	9	6	7	8	4	2	**1**
F8	Ave	1.0151E + 03	9.0607E + 02	1.0720E + 03	1.0121E + 03	9.7787E + 02	9.8528E + 02	9.8415E + 02	8.8648E + 02	**8.6491E + 02**
	Std	2.7821E + 01	3.3891E + 01	5.1363E + 01	5.4402E + 01	4.7014E + 01	3.8852E + 01	**1.6005E + 01**	2.1976E + 01	1.8627E + 01
	Rank	8	3	9	7	4	6	5	2	**1**
F9	Ave	2.0434E + 03	2.6993E + 03	1.2524E + 04	6.4863E + 03	5.2017E + 03	5.3498E + 03	1.3150E + 03	1.6802E + 03	**1.0254E + 03**
	Std	7.9405E + 02	1.0236E + 03	4.0753E + 03	2.0331E + 03	1.2788E + 03	1.2180E + 03	2.6733E + 02	4.9854E + 02	**1.7031E + 02**
	Rank	4	5	9	8	6	7	2	3	**1**
F10	Ave	7.5523E + 03	5.0409E + 03	7.2900E + 03	6.3907E + 03	7.4147E + 03	5.3007E + 03	7.6572E + 03	4.3919E + 03	**4.1699E + 03**
	Std	5.2829E + 02	1.5153E + 03	7.9821E + 02	1.1358E + 03	1.4405E + 03	7.3682E + 02	**3.2764E + 02**	7.7041E + 02	6.7062E + 02
	Rank	8	3	6	5	7	4	9	2	**1**
F11	Ave	1.6492E + 03	2.5100E + 03	1.0705E + 04	2.2445E + 03	3.6594E + 03	1.4657E + 03	1.2801E + 03	**1.2116E + 03**	1.2167E + 03
	Std	9.4403E + 02	1.1231E + 03	4.7657E + 03	1.6255E + 03	1.4077E + 03	1.6107E + 02	**2.8569E + 01**	3.4419E + 01	4.2580E + 01
	Rank	5	7	9	6	8	4	3	**1**	2
F12	Ave	1.7011E + 08	1.5293E + 08	3.9221E + 08	9.3439E + 07	1.0602E + 09	7.8352E + 08	1.0787E + 06	1.3478E + 06	**9.4918E + 05**
	Std	3.3818E + 08	2.4361E + 08	2.6303E + 08	1.3357E + 08	1.0521E + 09	1.7445E + 09	7.6194E + 05	9.2970E + 05	**7.5409E + 05**
	Rank	6	7	8	4	9	5	2	3	**1**
F13	Ave	9.6902E + 06	1.9491E + 07	9.9238E + 06	6.8224E + 06	3.9383E + 08	1.8625E + 08	2.1880E + 04	2.2859E + 04	**1.9619E + 04**
	Std	1.7212E + 07	7.0593E + 07	9.6768E + 06	1.4602E + 07	7.8300E + 08	5.0077E + 08	**1.2501E + 04**	1.7646E + 04	1.4357E + 04
	Rank	7	5	8	6	9	4	3	2	**1**
F14	Ave	1.2124E + 05	8.3096E + 05	2.2025E + 06	5.2592E + 05	7.6901E + 05	1.2799E + 04	**2.0708E + 03**	4.5507E + 04	8.7279E + 03
	Std	1.3663E + 05	1.0219E + 06	1.9348E + 06	8.1121E + 05	6.0931E + 05	1.9968E + 04	**7.1086E + 02**	4.0963E + 04	1.4263E + 04
	Rank	5	7	9	6	8	3	**1**	4	2
F15	Ave	1.8432E + 05	3.2506E + 06	7.7564E + 06	1.2254E + 05	1.9814E + 07	4.8513E + 05	**4.7956E + 03**	1.1471E + 04	9.3938E + 03
	Std	1.5258E + 05	1.0589E + 07	1.2257E + 07	1.7886E + 05	5.5242E + 07	2.3544E + 06	**3.0351E + 03**	1.1307E + 04	7.1380E + 03
	Rank	6	7	9	5	8	4	**1**	2	3
F16	Ave	3.0065E + 03	2.6830E + 03	4.3517E + 03	3.4626E + 03	3.2309E + 03	3.0706E + 03	3.0791E + 03	2.3620E + 03	**2.2223E + 03**
	Std	3.5721E + 02	3.8593E + 02	6.4138E + 02	4.7190E + 02	4.4818E + 02	3.4537E + 02	2.7340E + 02	2.8458E + 02	**2.5294E + 02**
	Rank	4	3	9	8	7	5	6	2	**1**
F17	Ave	2.1848E + 03	2.0649E + 03	2.8073E + 03	2.5703E + 03	2.2193E + 03	2.3563E + 03	2.0517E + 03	**1.9036E + 03**	1.9638E + 03
	Std	2.0652E + 02	1.7911E + 02	2.8044E + 02	2.9058E + 02	2.4063E + 02	2.1101E + 02	1.3946E + 02	**1.0047E + 02**	1.5404E + 02
	Rank	5	4	9	8	6	7	3	**1**	2
F18	Ave	1.8465E + 06	3.3832E + 06	1.8844E + 07	4.2742E + 06	4.6381E + 06	6.8720E + 05	1.3515E + 05	4.3126E + 05	**8.5208E + 04**
	Std	2.3545E + 06	5.4275E + 06	1.8396E + 07	4.4715E + 06	6.0051E + 06	2.3880E + 06	9.6449E + 04	3.3551E + 05	**8.4320E + 04**
	Rank	5	6	9	8	7	3	2	4	**1**
F19	Ave	2.8781E + 05	3.5901E + 06	2.4224E + 07	2.1003E + 06	3.3715E + 07	1.1862E + 06	**6.9974E + 03**	1.0652E + 04	8.3050E + 03
	Std	3.3468E + 05	9.2511E + 06	2.0353E + 07	3.7568E + 06	8.6537E + 07	4.2465E + 06	**4.7465E + 03**	1.3113E + 04	8.4239E + 03
	Rank	5	7	9	6	8	4	**1**	2	3
F20	Ave	2.5768E + 03	2.4967E + 03	2.9342E + 03	2.6349E + 03	2.6014E + 03	2.6165E + 03	2.4896E + 03	**2.2414E + 03**	2.3183E + 03
	Std	1.6036E + 02	2.0035E + 02	1.7350E + 02	2.2762E + 02	2.0689E + 02	1.9789E + 02	**1.1823E + 02**	1.2268E + 02	1.4028E + 02
	Rank	5	3	9	7	6	8	4	**1**	2
F21	Ave	2.5059E + 03	2.4153E + 03	2.6613E + 03	2.5512E + 03	2.4811E + 03	2.5476E + 03	2.4839E + 03	2.3783E + 03	**2.3578E + 03**
	Std	3.4771E + 01	4.0035E + 01	7.8459E + 01	4.7863E + 01	4.2932E + 01	5.2686E + 01	**1.5376E + 01**	2.6883E + 01	1.7049E + 01
	Rank	6	3	9	8	5	7	4	2	**1**
F22	Ave	5.2540E + 03	5.0046E + 03	8.0907E + 03	5.2658E + 03	6.2864E + 03	6.1952E + 03	**2.3098E + 03**	2.6899E + 03	2.8345E + 03
	Std	3.0611E + 03	2.6332E + 03	1.6420E + 03	2.8312E + 03	2.7064E + 03	1.5061E + 03	**3.8639E + 00**	1.2150E + 03	1.2213E + 03
	Rank	6	5	9	4	8	7	3	2	**1**
F23	Ave	2.9547E + 03	2.7773E + 03	3.1553E + 03	2.9984E + 03	2.8994E + 03	3.1389E + 03	2.8437E + 03	2.7447E + 03	**2.7026E + 03**
	Std	9.0947E + 01	4.6871E + 01	1.0617E + 02	8.2778E + 01	5.6420E + 01	1.0035E + 02	**1.9362E + 01**	3.1729E + 01	1.9820E + 01
	Rank	6	3	9	7	5	8	4	2	**1**
F24	Ave	3.1164E + 03	2.9636E + 03	3.2604E + 03	3.1849E + 03	3.1122E + 03	3.2879E + 03	3.0201E + 03	2.9122E + 03	**2.8711E + 03**
	Std	7.7149E + 01	6.3028E + 01	7.8813E + 01	9.8011E + 01	8.4044E + 01	1.2557E + 02	1.8503E + 01	2.6001E + 01	**1.8431E + 01**
	Rank	5	3	8	7	6	9	4	2	**1**
F25	Ave	2.9670E + 03	3.0191E + 03	3.2488E + 03	2.9834E + 03	3.2054E + 03	3.1669E + 03	2.9146E + 03	2.9108E + 03	**2.9013E + 03**
	Std	2.1203E + 01	5.7967E + 01	6.7074E + 01	6.7219E + 01	1.5090E + 02	2.9453E + 02	**1.8370E + 01**	2.1476E + 01	1.9987E + 01
	Rank	4	6	9	5	8	7	3	2	**1**
F26	Ave	5.3396E + 03	4.9316E + 03	8.5273E + 03	6.8200E + 03	6.2167E + 03	7.7202E + 03	5.1101E + 03	4.3607E + 03	**3.7101E + 03**
	Std	1.0261E + 03	**5.2119E + 02**	9.9390E + 02	9.9797E + 02	6.3604E + 02	1.6016E + 03	1.1301E + 03	9.5477E + 02	7.2447E + 02
	Rank	5	3	9	7	6	8	4	2	**1**
F27	Ave	3.2742E + 03	3.2771E + 03	3.4682E + 03	3.3228E + 03	3.3719E + 03	3.4395E + 03	3.2744E + 03	**3.2215E + 03**	3.2422E + 03
	Std	4.8196E + 01	3.7287E + 01	1.0485E + 02	5.8308E + 01	6.5796E + 01	1.6877E + 02	**1.1635E + 01**	1.5185E + 01	1.9479E + 01
	Rank	3	4	9	6	7	8	5	**1**	2
F28	Ave	3.3731E + 03	3.5408E + 03	4.0096E + 03	3.5812E + 03	3.8972E + 03	3.8298E + 03	3.2765E + 03	3.2490E + 03	**3.2146E + 03**
	Std	7.5565E + 01	1.8433E + 02	7.0779E + 02	4.2581E + 02	2.9637E + 02	7.0936E + 02	2.7248E + 01	2.3566E + 01	**1.5953E + 01**
	Rank	4	6	9	5	8	7	3	2	**1**
F29	Ave	4.1307E + 03	3.9857E + 03	5.4118E + 03	4.5032E + 03	4.2907E + 03	4.7138E + 03	3.9747E + 03	3.6589E + 03	**3.6222E + 03**
	Std	1.8598E + 02	2.3668E + 02	5.7728E + 02	4.3042E + 02	2.5064E + 02	6.8154E + 02	1.7100E + 02	1.5897E + 02	**1.5404E + 02**
	Rank	5	4	9	7	6	8	3	2	**1**
F30	Ave	2.8664E + 06	1.2400E + 07	7.6499E + 07	2.7043E + 06	4.5897E + 07	2.5597E + 06	1.1432E + 05	4.2458E + 04	**1.1953E + 04**
	Std	1.7538E + 06	9.9680E + 06	7.2003E + 07	4.0554E + 06	2.8686E + 07	2.9119E + 06	9.7527E + 04	9.5643E + 04	**6.1913E + 03**
	Rank	6	7	9	4	8	5	3	2	**1**
Ave.Rank		5.33	4.73	8.70	6.27	6.93	6.17	3.47	2.10	**1.30**
Friedman		5	4	9	7	8	6	3	2	**1**

As can be seen from the table, the data highlighted in bold is quite limited, indicating that QHSBOA exhibits a significant difference compared to these benchmark algorithms. This suggests that QHSBOA shows a significant difference when compared to the other metaheuristic algorithms.

### Time comparison analysis of QHSBOA and SBOA

**Fig. 7:**
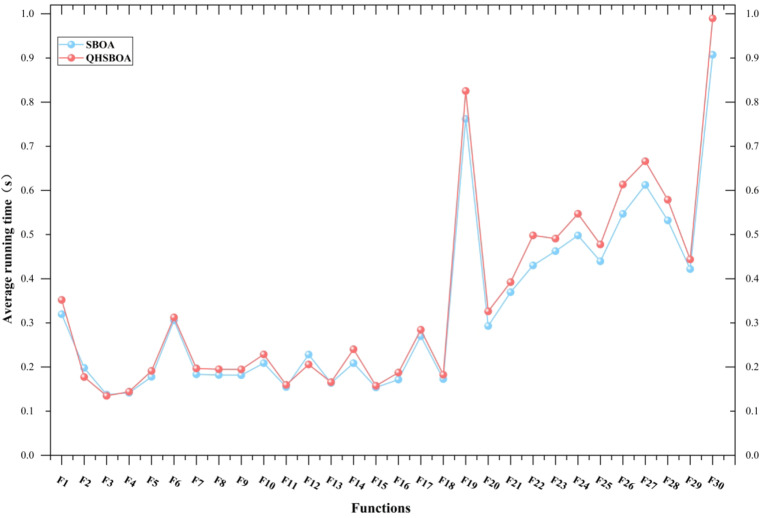
Comparison of computation time cost between QHSBOA and SBOA.

Based on the findings from the previous chapters, it is evident that the improved QHSBOA significantly outperforms the standard SBOA in overall performance. This section focuses on analyzing the differences in computational time costs between the two algorithms. To ensure fairness, the parameter settings for both QHSBOA and SBOA were standardized and kept consistent with those described earlier. Additionally, the average runtime for each algorithm was recorded over 30 independent executions. Figure 7 illustrates the average computational time (in seconds) required by both algorithms to solve each test function.

In the experimental results on the CEC 2017 benchmark set (30 dimensions), it can be observed that QHSBOA and SBOA exhibit almost identical execution times for unimodal functions and some relatively simple multimodal functions. However, when handling more complex hybrid functions, QHSBOA typically requires significantly more computational time than SBOA. This indicates that QHSBOA demonstrates higher computational efficiency in solving complex problems. Compared to the standard SBOA, QHSBOA not only incorporates a more efficient search strategy but also exhibits superior global search capability and local convergence speed. Overall, QHSBOA achieves higher solution accuracy than SBOA for most test functions, with the slight increase in runtime being negligible.

## Evaluate the proposed QHSBOA for diabetes classification

### KELM mathematical model

KELM (Kernel Extreme Learning Machine) is an enhancement of the traditional Extreme Learning Machine (ELM) that utilizes kernel functions to address classification and regression problems. By incorporating kernel methods, KELM extends the applicability of ELM, enabling it to handle nonlinear data. This improvement significantly enhances the model’s generalization performance and learning speed^72^.

The essence of ELM lies in determining a unique optimal solution by randomly initializing the input weights and biases of the hidden layer in the neural network, rather than through iterative adjustments. For $\:\text{n}$ t training samples $\:({\text{x}}_{\text{i}},{\text{t}}_{\text{i}})$ with $\:\text{K}$ hidden layer neurons and an activation function $\:\text{g}\left(\text{x}\right)$, where the input $\:{\text{x}}_{\text{i}}$ is an $\:\left(\text{n}\times\:1\right)$ dimensional training feature vector$\:{\text{x}}_{\text{i}}\in\:{\text{R}}^{\text{n}},{\text{t}}_{\text{i}}$ is an $\:\text{m}\times\:1$ dimensional target vector $\:{\text{t}}_{\text{i}}\in\:{\text{R}}^{\text{m}}$, t the output of the ELM is governed by a linear system. The mathematical model of Single Layer Feedforward Networks (SLFNs) is defined as follows^73,74^:

18 $$\:\begin{array}{c}{\text{O}}_{\text{j}}=\:\:\sum\:_{\text{i}=1}^{\text{K}}{{\upbeta\:}}_{\text{i}}\text{g}\left({\text{w}}_{\text{i}}\times\:{\text{x}}_{\text{i}}+{\text{b}}_{\text{i}}\right)\:\:\:\:\:\:i=\text{1,2},\dots\:,N;\:j=\text{1,2},\dots\:,N\end{array}$$

Where $\:{\text{O}}_{\text{j}}$ is the output vector of the $\:{\text{j}}^{\text{t}\text{h}}$ input, $\:{\text{b}}_{\text{i}}$ is the output weight vector connecting the $\:{\text{i}}^{\text{t}\text{h}}$ hidden neuron to the output layer, and $\:{\text{w}}_{\text{i}}$ is the weight vector connecting the $\:{\text{i}}^{\text{t}\text{h}}$ hidden neuron to the input layer. The term $\:{\text{w}}_{\text{i}}\times\:{\text{x}}_{\text{i}}$ represents the scalar product of $\:{\text{w}}_{\text{i}}$ and $\:{\text{x}}_{\text{i}}$, while $\:\text{g}\left({\text{w}}_{\text{i}}\times\:{\text{x}}_{\text{i}}+{\text{b}}_{\text{i}}\right)$ is the activation function of the $\:{\text{i}}^{\text{t}\text{h}}$ hidden neuron in the hidden layer.

The learning process of ELM aims to minimize the training error. When SLFNs can achieve zero error fitting for $\:\text{n}$ training samples, the following equation holds true: $\:{\sum\:}_{\text{i}=1}^{\text{N}}||{\text{t}}_{\text{i}}\times\:{\text{o}}_{\text{i}}||=0$, where $\:{{{\upbeta\:}}_{\text{i}}\text{w}}_{\text{i}}$ and $\:{\text{b}}_{\text{i}}$ exist such that:

19 $$\:\begin{array}{c}{\text{t}}_{\text{j}}={\text{o}}_{\text{j}}=\:\:\sum\:_{\text{i}=1}^{\text{K}}{{\upbeta\:}}_{\text{i}}\text{g}\left({\text{w}}_{\text{i}}\times\:{\text{x}}_{\text{i}}+{\text{b}}_{\text{i}}\right)\:\:\:\:\:\:j=\text{1,2},\dots\:,n\:\:\end{array}$$

20 $$\:\begin{array}{c}T=h\left(\text{x}\right)\beta\:=H\times\:\beta\end{array}$$

Where $\:\text{T}=[\text{t}_{1},\:\text{t}_{2},\:\ldots,{\:\text{t}}_{\text{n}}]^\text{T}$ and $\:\text{b}\:=\:[\text{b}_{1},\:\text{b}_{2},\:\ldots,\:\text{b}\text{K}]^\text{T}$. The function $\:\text{h}\left(\text{x}\right)$ serves as the feature mapping function, transforming the data from the input space into a $\:\text{K}$-dimensional feature space:

21 $$\:\begin{array}{c}H=h\left(\text{x}\right)={\left[\frac{{\text{h}}_{1}\left({\text{x}}_{\text{j}}\right)}{\begin{array}{c}\vdots \\\:{\text{h}}_{\text{K}}\left({\text{x}}_{\text{j}}\right)\end{array}}\right]}^{\text{T}}={\left[\begin{array}{c}g\left({\text{w}}_{1}\times\:{\text{x}}_{1}+{\text{b}}_{\text{i}}\right)\\\:\vdots\\\:g\left({\text{w}}_{1}\times\:{\text{x}}_{\text{N}}+{\text{b}}_{\text{i}}\right)\end{array}\begin{array}{c}\cdots\:\\\:\ddots\:\\\:\cdots\:\end{array}\begin{array}{c}g\left({\text{w}}_{\text{K}}\times\:{\text{x}}_{1}+{\text{b}}_{\text{i}}\right)\\\:\vdots\\\:g\left({\text{w}}_{\text{K}}\times\:{\text{x}}_{1}+{\text{b}}_{\text{i}}\right)\end{array}\right]}_{\text{N}\times\:\text{K}}\end{array}$$

The $\:\text{H}$ denote the output matrix of the hidden layer in the neural network, where the $\:{\text{i}}^{\text{t}\text{h}}$ c column corresponds to the output of the $\:{\text{i}}^{\text{t}\text{h}}$ hidden neuron for an $\:\text{n}$-dimensional input space. During the training process of Single-Layer Feedforward Networks (SLFNs), it is unnecessary to modify the input weights and biases of the hidden layer; these can be set arbitrarily. The network’s output $\:\text{b}$ can be calculated analytically. Under this premise, only the output weights require adjustment to optimize performance. The following rule is used to determine the output weights:

22 $$\:\begin{array}{c}{{\upbeta\:}}^{{\prime\:}}={\text{H}}^{+}\times\:T\end{array}$$

Where $\:{\text{H}}^{+}$ represent the Moore-Penrose pseudoinverse of the hidden layer output matrix $\:\text{H}$, which is computed using orthogonal projection as outlined below:

23 $$\:\begin{array}{c}H^{+}\:=\:H^{T}\left(HH^{T}\right)^{-1}\end{array}$$

The Moore-Penrose inverse approach ensures the minimum of all regular least squares solutions, resulting in a notable enhancement in learning speed and robust generalization performance.

The kernel-based Extreme Learning Machine was introduced by Huang et al.^75^ to improve the generalization ability of ELM, achieving better results than the least squares-based ELM. It is recommended to incorporate a regularization constant $\:\text{C}$ into the diagonal of $\:\text{H}^\text{T}\text{H}$ during the computation of the output weights $\:\text{b}$, as described below:

24 $$\:\begin{array}{c}\beta\:={\text{H}}^{\text{T}}{\left(\frac{\text{I}}{\text{C}}+\text{H}{\text{H}}^{\text{T}}\right)}^{-1}T\end{array}$$

Where $\:\text{C}$ is the penalty parameter and $\:\text{I}$ is the identity matrix. Therefore, the definition of the output function can be expressed as:

25 $$\:\begin{array}{c}F\left(\text{x}\right)={\text{h}\left(\text{x}\right)\text{H}}^{\text{T}}{\left(\frac{\text{I}}{\text{C}}+\text{H}{\text{H}}^{\text{T}}\right)}^{-1}T\end{array}$$

The KELM can subsequently be derived as follows:

26 $$\:\begin{array}{c}{{\Omega\:}}_{\text{E}\text{L}\text{M}}=H{\text{H}}^{\text{T}}:{\:{\Omega\:}}_{{\text{E}\text{L}\text{M}}_{\text{i},\text{j}}}={\text{h}\left({\text{x}}_{\text{i}}\right)}^{2}=K\left({\text{x}}_{\text{i}},{\text{x}}_{\text{i}}\right)\end{array}$$

In this equation $\:\text{K}\left({\text{x}}_{\text{i}},{\text{x}}_{\text{j}}\right)$ represents the kernel matrix derived from the hidden layer outputs.

27 $$\:\begin{array}{c}F\left(\text{x}\right)={\left[\begin{array}{c}K\left(\text{x},{\text{x}}_{1}\right)\\\:\dots\:\\\:K\left(\text{x},{\text{x}}_{\text{n}}\right)\end{array}\right]}^{\text{T}}\times\:{\left(\frac{\text{I}}{\text{C}}+{{\Omega\:}}_{\text{E}\text{L}\text{M}}\right)}^{-1}T\end{array}$$

**Fig. 8:**
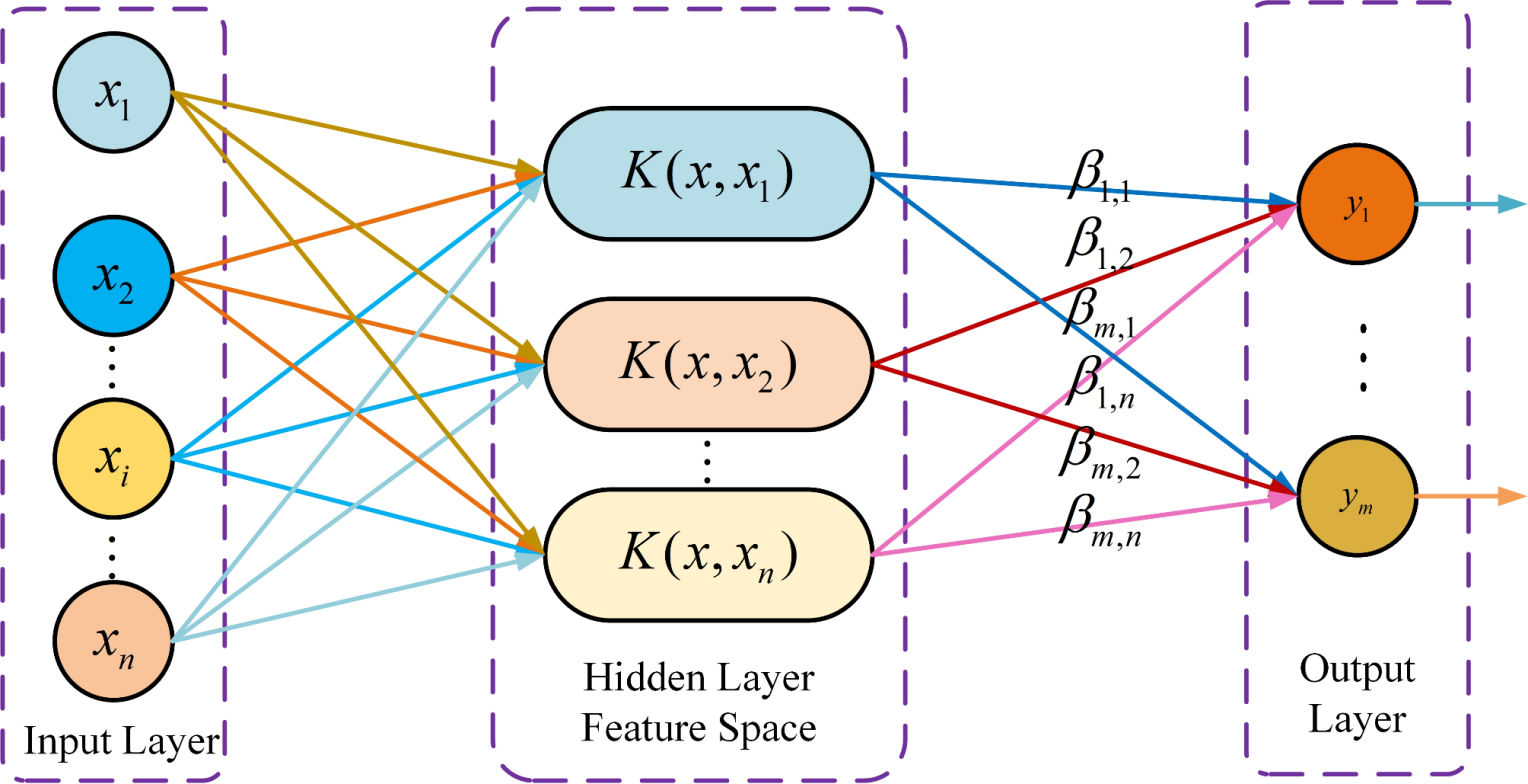
The diagrammatic representation of the KELM model’s architecture.

KELM, which implements ELM using this kernel-based approach, provides enhanced stability and generalization performance. Figure 8 provides a concise overview of the KELM model’s architecture, in which the kernel function substitutes the conventional feature mapping function, enabling the transition from the input space to the feature space. As a result, the neural network’s output is no longer dependent on the hidden layer’s feature mapping; instead, it is directly dictated by the kernel function. Additionally, the dimensionality of the feature mapping in the hidden layer is no longer predetermined.

In this research, the Gaussian radial basis function serves as the kernel function for KELM, defined by the equation below:

28 $$\:\begin{array}{c}K\left({\text{x}}_{\text{i}},{\text{x}}_{\text{j}}\right)={\text{e}}^{\frac{{||{\text{x}}_{\text{i}}-{\text{x}}_{\text{j}}||}^{2}}{2{{\upsigma\:}}^{2}}}\end{array}$$

The penalty parameter $\:\text{C}$ and the kernel parameter $\:\text{c}$ are essential in model development. The penalty parameter $\:\text{C}$ governs the trade-off between reducing fitting errors and managing model complexity. The kernel parameter $\:\text{c}$ defines the nonlinear transformation from the input space to a particular high-dimensional feature space within the hidden layer. Generally, to enhance the performance of KELM, both of these key parameters can be optimally selected using appropriate optimization algorithms.

### KELM based on QHSBOA

In this section, we introduce an improved KELM model, referred to as QHSBOA-KELM. This model is based on the previously discussed QHSBOA algorithm and is capable of adaptively optimizing two key parameters of KELM: kernel penalty parameter $\:\text{C}$ and the kernel parameter $\:\text{c}$. The ranges for these optimized parameters are detailed in Tables  5. The model primarily consists of two modules: the first module optimizes the parameters within the internal loop, while the second module evaluates the classification features in the external loop. During the parameter optimization process, QHSBOA is employed to dynamically adjust the parameters of KELM, applying the best parameters obtained to the KELM classification model to address the classification problem. The design of the fitness function f takes into account the accuracy of the classification.

**Table 4:** P-value on CEC2017 (dim = 30).

Function	PSO	GWO	WOA	DBO	GJO	BKA	CPO	SBOA
F1	3.0199E-11	3.0199E-11	3.0199E-11	3.0199E-11	3.0199E-11	3.0199E-11	3.0199E-11	6.5277E-08
F2	3.0199E-11	3.0199E-11	3.0199E-11	3.0199E-11	3.0199E-11	3.0199E-11	3.0199E-11	2.8716E-10
F3	3.0199E-11	3.0199E-11	3.0199E-11	3.0199E-11	3.0199E-11	3.0199E-11	3.0199E-11	3.0199E-11
F4	1.9568E-10	7.3803E-10	3.0199E-11	4.9752E-11	3.0199E-11	3.6897E-11	1.7666E-03	**5.1877E-02**
F5	3.0199E-11	4.5043E-11	3.0199E-11	3.0199E-11	3.0199E-11	3.0199E-11	3.0199E-11	7.6950E-08
F6	3.0199E-11	3.3384E-11	3.0199E-11	3.0199E-11	3.0199E-11	3.0199E-11	1.4067E-04	6.5277E-08
F7	3.0199E-11	3.6897E-11	3.0199E-11	3.0199E-11	3.0199E-11	3.0199E-11	3.0199E-11	7.3891E-11
F8	3.0199E-11	2.7829E-07	3.0199E-11	3.0199E-11	3.3384E-11	3.0199E-11	3.0199E-11	3.7704E-04
F9	1.9568E-10	7.3891E-11	3.0199E-11	3.0199E-11	3.0199E-11	3.0199E-11	6.0459E-07	1.0105E-08
F10	3.0199E-11	2.3243E-02	3.0199E-11	8.9934E-11	3.3384E-11	3.5201E-07	3.0199E-11	2.8230E-02
F11	3.0199E-11	3.0199E-11	3.0199E-11	3.0199E-11	3.0199E-11	4.9752E-11	9.8329E-08	1.1188E-02
F12	3.0199E-11	3.0199E-11	3.0199E-11	3.0199E-11	3.0199E-11	2.1544E-10	**5.1060E-01**	4.9013E-02
F13	3.0199E-11	3.0199E-11	3.0199E-11	4.5043E-11	3.0199E-11	3.0199E-11	4.7108E-02	**5.9231E-02**
F14	6.7220E-10	1.6132E-10	3.3384E-11	2.6099E-10	1.2057E-10	**1.2597E-01**	**2.0095E-01**	2.3768E-07
F15	3.0199E-11	4.5043E-11	3.0199E-11	1.2023E-08	3.0199E-11	1.6947E-09	7.2884E-03	4.6956E-02
F16	1.2870E-09	7.0430E-07	3.0199E-11	4.5043E-11	7.3891E-11	4.1997E-10	8.9934E-11	4.5146E-02
F17	5.9706E-05	4.3584E-02	4.9752E-11	1.1737E-09	1.6351E-05	5.4617E-09	2.2360E-02	**1.2597E-01**
F18	1.3289E-10	1.3289E-10	3.0199E-11	1.9568E-10	2.1544E-10	3.3679E-04	6.9125E-04	3.9648E-08
F19	1.7769E-10	3.0199E-11	3.0199E-11	6.0658E-11	3.0199E-11	3.1589E-10	**9.0000E-01**	**7.0516E-02**
F20	2.1959E-07	7.1988E-05	3.0199E-11	2.0283E-07	9.5332E-07	1.7294E-07	3.3681E-05	3.7782E-02
F21	3.0199E-11	8.1014E-10	3.0199E-11	3.0199E-11	4.0772E-11	3.3384E-11	3.0199E-11	1.9527E-03
F22	7.0881E-08	1.4733E-07	1.3289E-10	2.3897E-08	1.3111E-08	6.7220E-10	6.7650E-05	9.8834E-03
F23	9.9186E-11	7.3803E-10	3.0199E-11	3.0199E-11	3.0199E-11	3.0199E-11	3.0199E-11	8.8411E-07
F24	3.0199E-11	2.3715E-10	3.0199E-11	3.0199E-11	3.0199E-11	3.0199E-11	3.0199E-11	1.2541E-07
F25	7.3891E-11	3.0199E-11	3.0199E-11	3.0811E-08	3.0199E-11	3.0199E-11	1.1738E-03	2.3243E-02
F26	1.2860E-06	1.6947E-09	3.0199E-11	1.7769E-10	3.0199E-11	2.8716E-10	1.2860E-06	1.4067E-04
F27	1.1738E-03	8.1465E-05	3.0199E-11	1.2870E-09	3.6897E-11	6.6955E-11	7.6950E-08	3.8307E-05
F28	3.0199E-11	3.0199E-11	3.0199E-11	3.0199E-11	3.0199E-11	3.0199E-11	8.1014E-10	3.0103E-07
F29	6.6955E-11	9.0632E-08	3.0199E-11	9.9186E-11	3.6897E-11	5.4941E-11	4.5726E-09	**3.8710E-01**
F30	3.0199E-11	3.0199E-11	3.0199E-11	3.6897E-11	3.0199E-11	3.0199E-11	4.9752E-11	2.0058E-04

**Table 5:** P-value on CEC2017 (Dim = 30).

Parameter	Search range
Penalty parameter $\:\left(\text{C}\right)$	$\:\left[\text{1,20}\right]$
Kernel parameter $\:\left(\text{c}\right)$	$\:\left[\text{1,20}\right]$

29 $$\:\begin{array}{c}f=avgAcc={\sum\:}_{\text{i}}^{\text{K}}\frac{\text{t}\text{e}\text{s}\text{t}{\text{A}\text{c}\text{c}}_{\text{i}}}{\text{K}}\end{array}$$

In this context, $\:\text{a}\text{v}\text{g}\text{A}\text{c}\text{c}$ represents the average test accuracy obtained by the KELM classifier during parameter optimization through 10-fold cross-validation.

Parameter range setting.

**Fig. 9:**
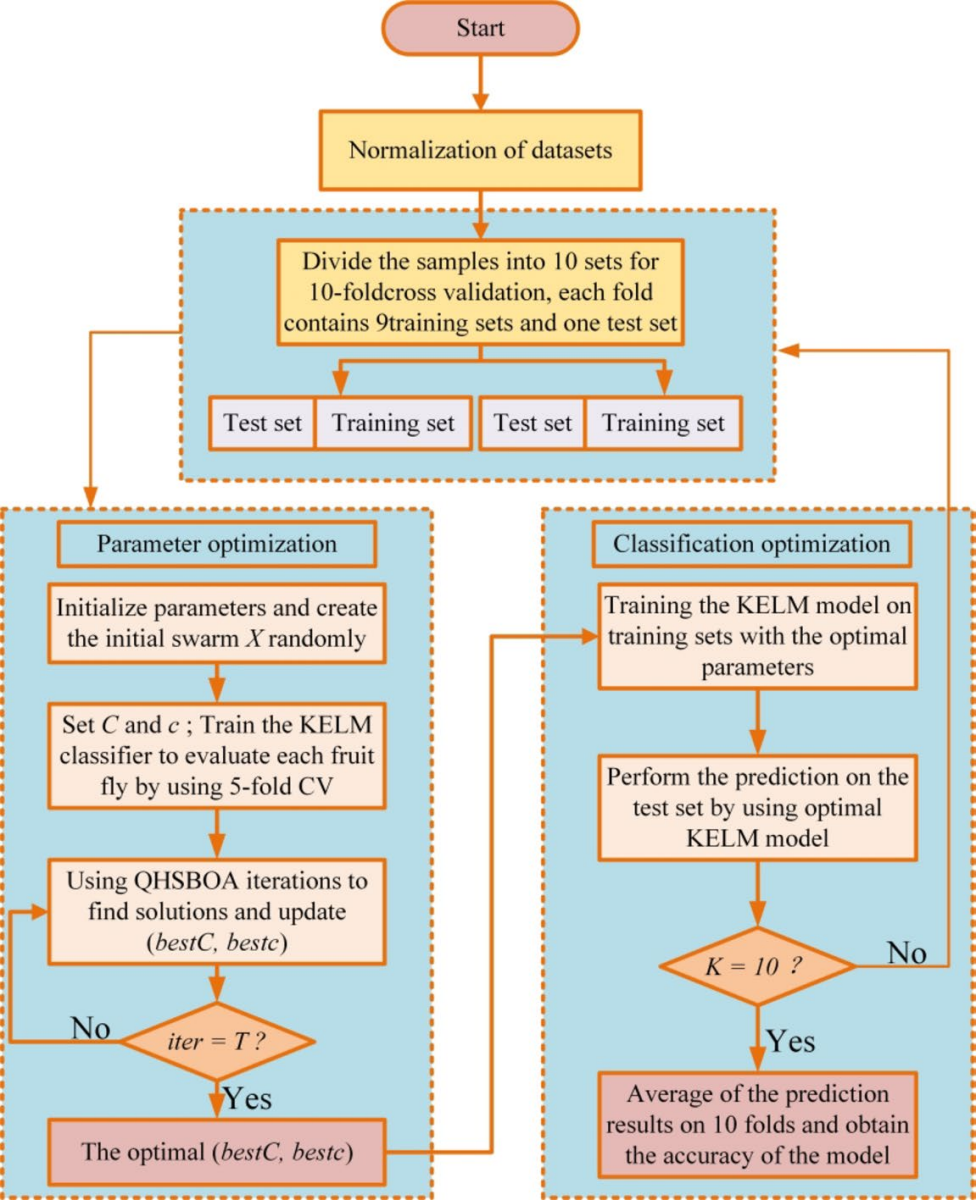
Diabetes classification prediction process based on QHSBOA-KELM.

The overall framework of the QHSBOA-based KELM diabetes classification prediction model is illustrated in Fig. 9. To ensure the reliability and unbiased nature of the study results, a 10-fold cross-validation (CV) was employed to assess the classifier’s performance, while a 5-fold CV was used internally to optimize the two classification parameters. In each experiment, 7 out of 10 subsets were selected as training samples, with the remaining subsets used as the test set. Additionally, the fifth subset extracted from specific samples served as the validation dataset. This experimental design aids in providing an unbiased estimate of generalization accuracy, ensuring the credibility of the results. Notably, the dataset was divided using a stratified strategy, ensuring that samples were drawn in proportion to the number of diabetes patients in each fold. Considering that the randomness of sampling might impact the results, a single round of 10-fold CV was insufficient to achieve optimal classification accuracy. Therefore, each method underwent 30 rounds of 10-fold CV, and the average of these 30 results was taken as the final evaluation outcome.

The basic principle of 10 - fold cross - validation is as follows: The dataset is divided into 10 mutually exclusive subsets of similar size, and then 10 rounds of training and testing are carried out. In each round, one subset is selected as the test set, and the remaining 9 subsets are combined to form the training set. After using the training set to train the model, the performance of the model is evaluated with the test set, such as calculating metrics like accuracy and mean squared error. After 10 rounds of operations, the 10 performance metrics obtained are combined (usually by taking the average), and this is used as the final performance evaluation result of the model. In this way, the generalization ability of the model can be evaluated more stably and comprehensively, and the evaluation bias caused by a single data division can be reduced.

The dataset used in this study is the widely recognized Pima Indian Diabetes Dataset (PIDD), which is commonly employed in various research studies. This dataset originates from the National Institute of Diabetes and Digestive and Kidney Diseases (NIDDK), a prestigious medical research institution, ensuring the data’s high reliability and credibility. The diabetes dataset contains 768 records, with each sample having 8 features and 1 corresponding label. These 8 features are: number of pregnancies, glucose level, blood pressure, skin thickness, insulin level, body mass index (BMI), family history of diabetes, and age. These features are critical in determining the likelihood of developing diabetes. Regarding the labels, individuals diagnosed with diabetes are marked as “1”, while those without diabetes are labeled as “0”. The dataset includes 268 diabetic patients and 500 non-diabetic individuals.

There are some missing values in the data, and in this study, the missing values are imputed using mean substitution. Additionally, the values of these features are normalized to the [0,1] range, which enhances the model’s convergence speed and performance. This normalization allows the deep learning model to better capture the relationships between features during training, enabling more efficient learning of the patterns in the data, thereby improving the overall prediction accuracy.

### Classification prediction of diabetes

#### Model evaluation index

The most commonly used metrics for evaluating multi-class quality were employed to assess the performance of the proposed method. The model evaluation metrics used in this study include Accuracy (ACC), Matthews Correlation Coefficient (MCC), Sensitivity, and Specificity^76–78^. The definitions of these four metrics are as follows:

30 $$\begin{aligned}ACC &= \frac{1}{n}\sum_{i = 1}^{n}\frac{TP_{i}+TN_{i}}{TP_{i}+FP_{i}+FN_{i}+TN_{i}}\times 100\%\\MCC &= \frac{1}{n}\sum_{i = 1}^{n}\frac{TP_{i}\times TN_{i}-FP_{i}\times FN_{i}}{\sqrt{(TP_{i}+FP_{i})\times(TP_{i}+FN_{i})\times(TN_{i}+FP_{i})\times(TN_{i}+FN_{i})}}\times 100\%\\Sensitivity &= \frac{1}{n}\sum_{i = 1}^{n}\frac{TP_{i}}{TP_{i}+FN_{i}}\times 100\%\\Specificity &= \frac{1}{n}\sum_{i = 1}^{n}\frac{TN_{i}}{TN_{i}+FP_{i}}\times 100\%\end{aligned}$$

In this context, $\:{\text{T}\text{P}}_{\text{i}}$ represents the number of true positives, which are the instances accurately classified as “positive”. $\:{\text{T}\text{N}}_{\text{i}}$ denotes the number of true negatives, indicating the instances correctly identified as “positive”. Similarly, $\:{\text{F}\text{N}}_{\text{i}}$ refers to the number of false positives, which are negative instances misclassified as “positive”. $\:{\text{F}\text{P}}_{\text{i}}$ signifies the number of false negatives, or positive instances incorrectly labeled as “negative”. The Matthews correlation coefficient $\:\text{M}\text{C}\text{C}$ is a well-established metric that reflects the correlation between observed and predicted values. The $\:\text{M}\text{C}\text{C}$ value indicates the model’s effectiveness in addressing imbalanced datasets. Sensitivity measures the classification method’s capability to detect positive cases, while specificity measures its ability to identify negative cases.

#### Classification prediction experiment results and analysis

**Fig. 10:**
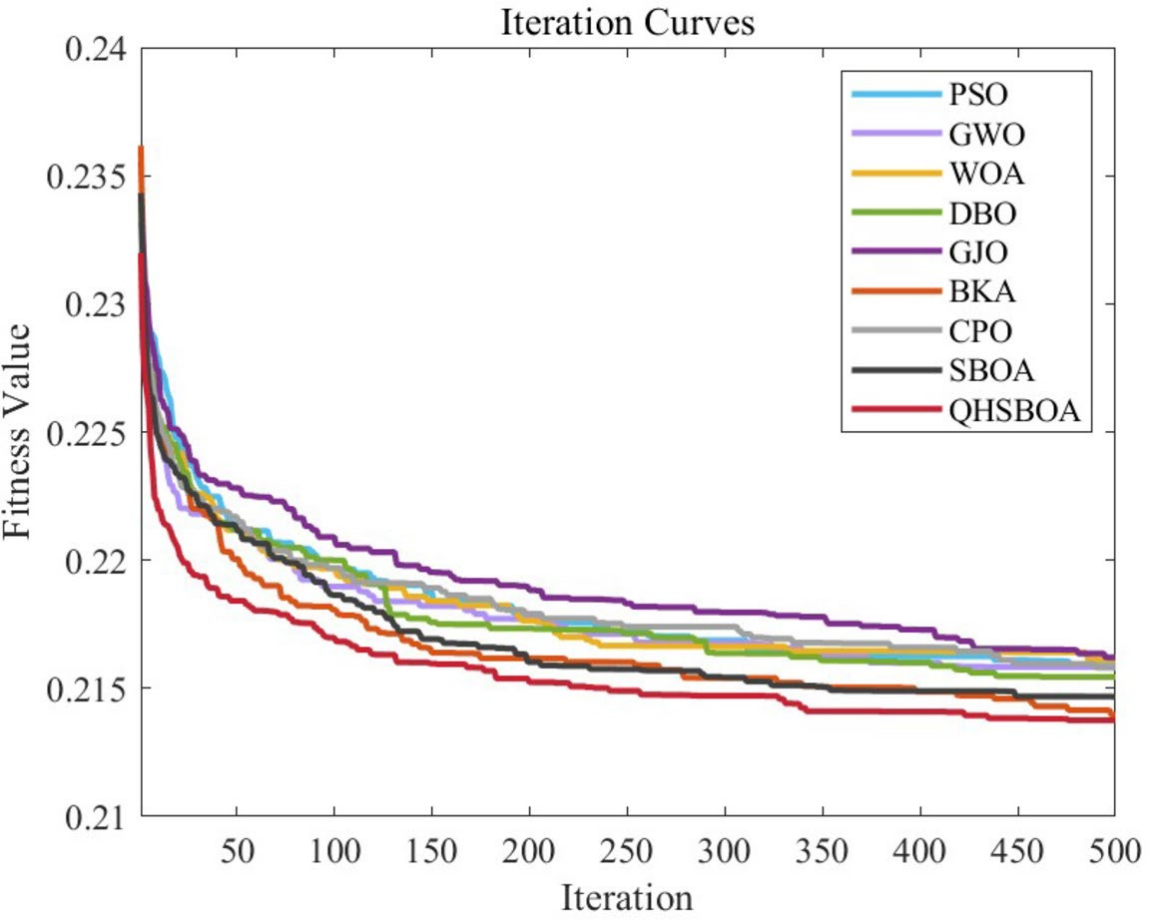
Iteration curves of KELM parameters optimized by different algorithms.

**Fig. 11:**
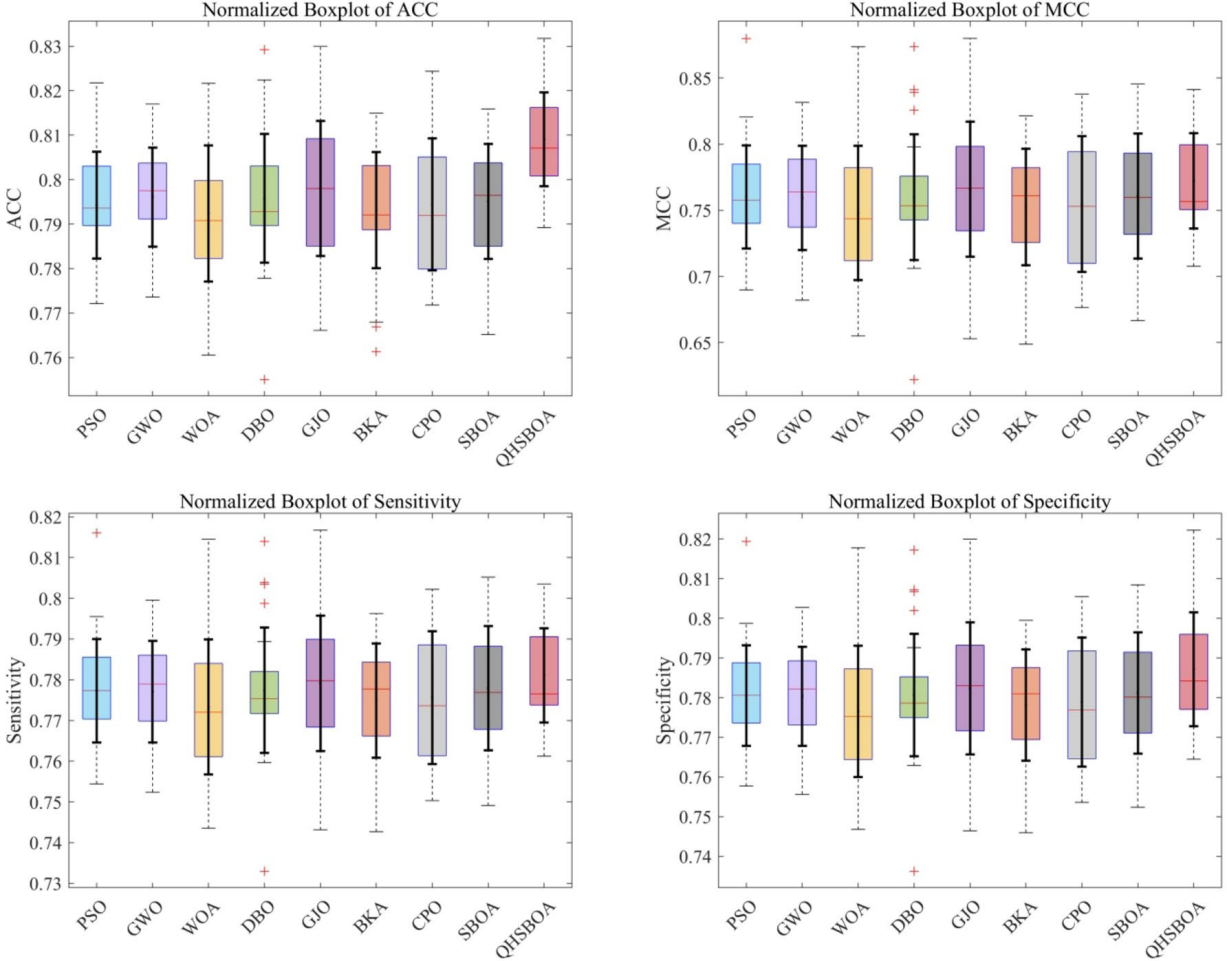
Box plot of evaluation indicators of QHSBOA-KELM and its comparison algorithm on diabetes data set.

Table 6 presents the classification accuracy of different methods, while Fig. 10 illustrates the convergence curves of the fitness function for KELM classification predictions optimized by various algorithms. Figure 11 displays a box plot comparing the classification prediction results of the optimized KELM across different algorithms on the diabetes dataset.

**Table 6:** Comparison of classification accuracy of different algorithms.

Method	PSO-KELM	GWO-KELM	WOA-KELM	DBO-KELM	GJO-KELM	BKA-KELM	CPO-KELM	SBOA-KELM	QHSBOA-KELM
Classification accuracy (%)	78.45%	78.49%	78.07%	78.24%	78.50%	78.40%	78.06%	78.43%	78.63%
F1-score	72.03	72.53	71.11	71.85	72.53	72.27	72.21	72.37	72.98
Recall	70.97	71.20	70.34	70.04	70.97	70.22	70.89	70.43	71.28

**Fig. 12:**
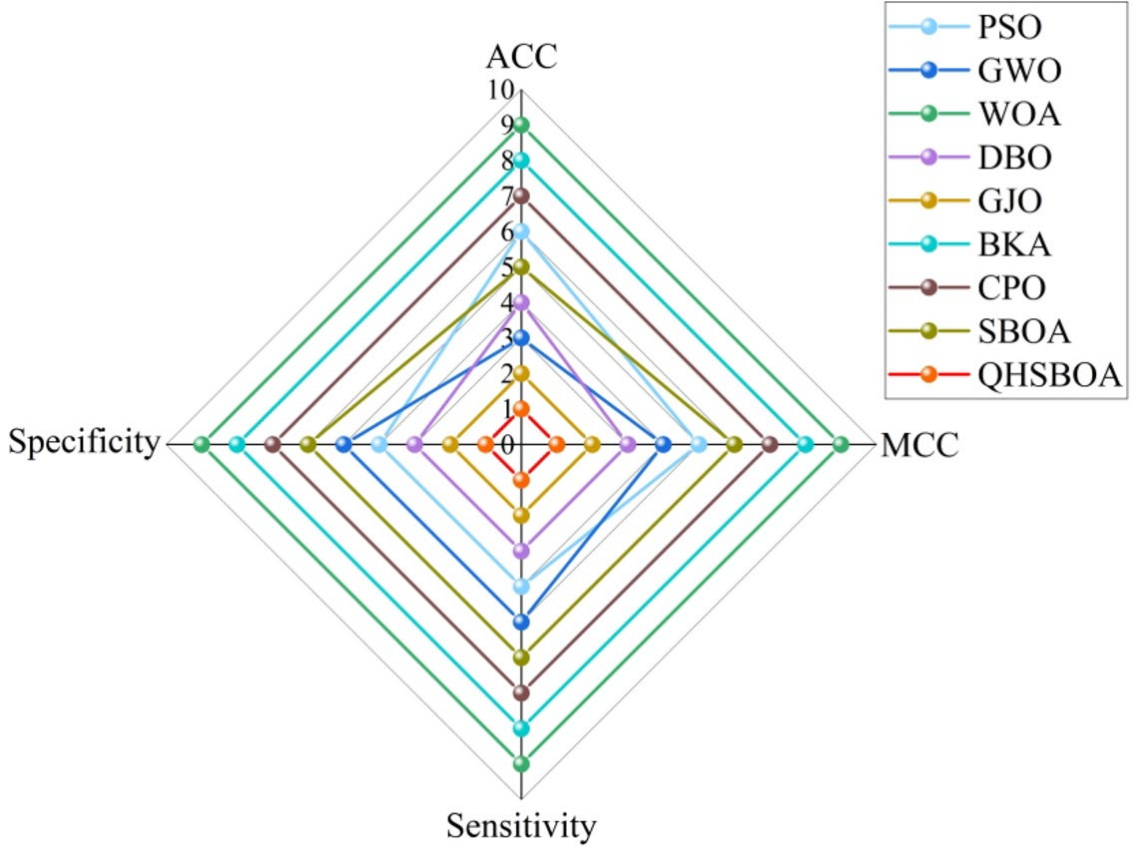
Different index rankings obtained by different calculations.

From Table 5, it is evident that the accuracy of the QHSBOA-KELM diabetes classification model is 78.63%, which is higher than the 78.43% achieved by SBOA-KELM, representing an improvement of 0.2%. Compared to PSO-KELM, GWO-KELM, BKA-KELM, and CPO-KELM, the accuracy is also increased by 0.18%, 0.14%, 0.23%, and 0.57%, respectively.

Although the performance improvement of QHSBOA-KELM is relatively modest compared to other advanced algorithms, this does not diminish its crucial role in the field of diabetes prediction. In the prevention and treatment of diabetes, predictive accuracy plays a pivotal role. For patients, accurate predictions mean being able to assess their health status at an early stage, enabling them to take timely preventive measures and modify unhealthy lifestyle habits, thereby effectively reducing the risk of developing diabetes. Even a slight improvement in predictive accuracy can provide patients with a valuable time window, allowing for intervention before the disease worsens. This has a significant impact on improving the quality of life and health of patients, highlighting the unique and important value of QHSBOA-KELM in the domain of diabetes prediction.

This minor improvement, when applied to diabetes screening, can be used as a routine check-up to screen high-risk individuals in community health centers, while enhancing diagnostic efficiency and accuracy in hospital endocrinology departments. However, its limitations are apparent. If the dataset is limited in size, it may fail to cover the full spectrum of diabetes patient characteristics, potentially missing rare cases. Regional bias also affects its accuracy, as lifestyle habits, dietary structures, and genetic backgrounds vary across different regions. Models trained on data from a single region may not perform well in other areas. Therefore, it is necessary to collect diverse data from various regions to ensure the model can be effectively applied across different populations.

Figure 10 illustrates that the proposed QHSBOA demonstrates superior performance in both convergence speed and solution accuracy. Specifically, QHSBOA exhibits a rapid decline in the convergence curve during the early iterations, outperforming all comparison algorithms. In contrast, the original SBOA shows a slower convergence speed in the early stages and ultimately achieves lower solution accuracy, which is not as high as that of the recently proposed BKA. This can be attributed to the fact that the PSO search mechanism introduces additional randomness to stimulate a broader global search, effectively avoiding local optima. The dynamic boundary adjustment based on the optimal individual makes the search process more flexible, allowing the algorithm to conduct a more detailed search in the vicinity of the current best solution. This enhances both the efficiency and quality of the optimization process, thereby accelerating the algorithm’s convergence speed.

From Fig. 11, it is evident that QHSBOA-KELM achieves the best performance across the four evaluation metrics: ACC, MCC, Sensitivity, and Specificity. In contrast, the values generated by CPO-KELM are the lowest for all four metrics, indicating that this method is less effective for diabetes classification predictions. In terms of stability, QHSBOA-KELM ranks second, just behind GWO-KELM, while BKA-KELM and CPO-KELM exhibit the poorest stability, and SBOA-KELM also demonstrates weak stability. Regarding solution accuracy, QHSBOA-KELM is the best among all models, with SBOA-KELM only ranking third. Additionally, SBOA-KELM performs worse than both GWO-KELM and GJO-KELM across all four metrics.

From Fig. 12, it can be observed that the QHSBOA-based KELM method proposed in this paper ranks first in all four evaluation metrics. In contrast, the standard SBOA-based KELM method ranks only fifth in ACC, and sixth in MCC, Sensitivity, and Specificity. This demonstrates the effectiveness of the proposed method, which achieves better optimization and prediction results in diabetes classification.

In summary, the QHSBOA-KELM classifier outperforms other comparative models due to the powerful optimization capabilities of the proposed QHSBOA. The significant differences in results produced by SBOA-KELM on the diabetes dataset reveal that its performance is unstable. This further highlights the strong optimization performance of QHSBOA, which surpasses that of other comparison algorithms. It indicates that the QHSBOA-KELM model demonstrates high stability and accuracy in the binary classification problem of diabetes prediction, as well as strong classification ability. Moreover, it underscores the effectiveness of the three introduced improvement strategies, which enhance the overall performance of SBOA.

## Summary and prospect

To test the effectiveness of the proposed QHSBOA, a comparative analysis was conducted between QHSBOA and several popular metaheuristic algorithms, as well as the standard SBOA, using the CEC2017 benchmark functions. The experimental results confirmed that the improved QHSBOA with its three strategies exhibits outstanding performance, outperforming the original SBOA and other comparison algorithms in the majority of functions. Furthermore, various KELM diabetes classification prediction models based on optimization algorithms were developed to predict diabetes risk based on user-input feature data. This model utilized the Pima Indians Diabetes Dataset and employed cross-validation along with optimization algorithms to identify the optimal parameter combinations for the KELM prediction model. Finally, the prediction performance of the model was evaluated using the test set. Analysis of metrics such as ACC, MCC, Sensitivity, and Specificity revealed that the QHSBOA-KELM model achieved the highest accuracy in prediction results, demonstrating good classification capability and high stability in predicting diabetes. Therefore, it can be concluded that the applicability of QHSBOA-KELM has been expanded, and the proposed method can assist healthcare practitioners in making accurate assessments of patients, thereby facilitating the development of effective prevention and treatment strategies.

In summary, owing to the robust optimization capabilities of the proposed QHSBOA, the QHSBOA-KELM classifier outperforms other comparative models. The significant differences generated by SBOA-KELM on the diabetes dataset indicate its relatively stable performance. This further underscores the powerful optimization performance of QHSBOA, surpassing other comparative algorithms. These findings demonstrate that the QHSBOA-KELM model exhibits high stability and accuracy, along with strong classification capabilities, in the binary classification problem of diabetes prediction.

In future research, we aim to integrate QHSBOA with other algorithms for collaborative optimization, fully leveraging the strengths of each algorithm to further enhance the accuracy and robustness of problem-solving. We plan to introduce techniques such as machine learning and deep learning to improve the adaptive and self-learning capabilities of QHSBOA, enabling it to adapt to changes in various problems and environments. This will allow the algorithm to learn from data and adjust its parameters and strategies, thereby improving its effectiveness and adaptability. Furthermore, the proposed QHSBOA-KELM can be applied to address other classification problems in real life, such as cancer risk prediction and fault diagnosis.

## Data Availability

All data generated or analysed during this study are included in this published article.

## References

[1] Shen, Y. et al. Sep., SGLT2 inhibitor empagliflozin downregulates miRNA-34a-5p and targets GREM2 to inactivate hepatic stellate cells and ameliorate non-alcoholic fatty liver disease-associated fibrosis, (in eng), *metabolism*, **146**, p. 155657, (2023). 10.1016/j.metabol.2023.155657 37422021

[2] [Clinical guidelines for prevention and treatment of. Type 2 diabetes mellitus in the elderly in China (2022 edition)], (in chi). *Zhonghua Nei Ke Za Zhi*, **61**, 1, pp. 12–50, 2022. 10.3760/cma.j.cn112138-20211027-00751 34979769

[3] Zhao, X., Zhang, Y., Yang, Y. & Pan, J. Diabetes-related avoidable hospitalisations and its relationship with primary healthcare resourcing in China: a cross-sectional study from Sichuan Province, (in eng). *Health Soc. Care Community*. **30** (4), e1143–e (2022). 10.1111/hsc.13522 34309097

[4] Varma, K. V. S. R. P., Rao, A. A., Sita Maha, T., Lakshmi & Nageswara Rao, P. V. A computational intelligence approach for a better diagnosis of diabetic patients, *Computers & Electrical Engineering*, vol. 40, no. 5, pp. 1758–1765, /07/01/ 2014. (2014).

[5] Sahebi, H. R. & Ebrahimi, S. and I. J. A. i. C. S. a. I. J. Ashtian, a fuzzy classifier based on modified particle swarm optimization for diabetes disease diagnosis, **4**, 3, pp. 11–17, (2015).

[6] Sisodia, D., Sisodia, D. S. J. P. c. s. Prediction of diabetes using classification algorithms. **132**, 1578–1585 (2018).

[7] Maniruzzaman, M., Rahman, M. J. & Ahammed, M. M. J. H. i. s. Abedin, and systems. Classification and prediction of diabetes disease using machine learning paradigm. **8**, 1–14 (2020). 10.1007/s13755-019-0095-z PMC6942113 31949894

[8] Azad, C. et al. Prediction model using SMOTE, genetic algorithm and decision tree (PMSGD) for classification of diabetes mellitus, pp. 1–19, (2022).

[9] Shankaracharya, D. O., Vidyarthi, A. S., o., S. J. T. R. & Samanta, D. S. Computational intelligence in early diabetes diagnosis: a review, **7**, no. 4, (2010). 10.1900/RDS.2010.7.252 PMC3143540 21713313

[10] Varma, K. V., Rao, A. A., Lakshmi, T. S. M., Rao, P. N. J. C. & Engineering, E. A computational intelligence approach for a better diagnosis of diabetic patients, **40**, 5, pp. 1758–1765, (2014).

[11] Huang, H., Wu, N., Liang, Y., Peng, X. & Shu, J. SLNL: A novel method for gene selection and phenotype classification, **37**, 9, pp. 6283–6304, (2022).

[12] Wei, Y. et al. Exploring the causal relationships between type 2 diabetes and neurological disorders using a mendelian randomization strategy, **103**, 46, p. e40412, (2024). 10.1097/MD.0000000000040412 PMC11576012 39560586

[13] Polat, K. & Güneş, S. and A. J. E. s. w. a. Arslan, a cascade learning system for classification of diabetes disease: generalized discriminant analysis and least square support vector machine, **34**, 1, pp. 482–487, (2008).

[14] Beloufa, F. M. A. J. C. m. Chikh, and p. i. biomedicine, Design of fuzzy classifier for diabetes disease using Modified Artificial Bee Colony algorithm, vol. 112, no. 1, pp. 92–103, (2013). 10.1016/j.cmpb.2013.07.009 23932385

[15] Han, X. H. et al. A Novel Power Transformer Fault diagnosis model based on Harris-hawks-optimization Algorithm Optimized Kernel Extreme Learning Machine. *J. Electr. Eng. Technol.*, **17**, 3, pp. 1993–2001, 2022.

[16] Lu, H. J., Du, B. J., Liu, J. Y., Xia, H. X. & Yeap, W. K. A kernel extreme learning machine algorithm based on improved particle swam optimization. *Memetic Comput.* **9** (2), 121–128 (2017).

[17] Hu, Q. et al. Time-Frequency Fusion Features-Based GSWOA-KELM Model for Gear Fault Diagnosis, *Lubricants*, vol. 12, no. 1, Jan Art. no. 10. (2024).

[18] He, C. M., Xu, F. H., Liu, Y. Q. & Zheng, J. H. A fast kernel extreme learning machine based on conjugate gradient. *Network-Computation Neural Syst.* **29**, 1–4 (2018). 10.1080/0954898X.2018.1562247 30688136

[19] Chen, X. Y., Dong, X. L. & Shi, L. Short-term power load forecasting based on I-GWO-KELM algorithm, in *2nd International Conference on Computer Science Communication and Network Security (CSCNS)*, Sanya, PEOPLES R CHINA, vol. 336, 2021. (2020).

[20] He, W. M., Xie, Y. Q., Lu, H. X., Wang, M. J. & Chen, H. L. Predicting Coronary Atherosclerotic Heart Disease: An Extreme Learning Machine with Improved Salp Swarm Algorithm, *Symmetry-Basel*, vol. 12, no. 10, Oct Art. no. 1651. (2020).

[21] Xia, J. F. et al. Feb., Evolving kernel extreme learning machine for medical diagnosis via a disperse foraging sine cosine algorithm. *Comput. Biol. Med.*, **141**, (2022). Art. 105137. 10.1016/j.compbiomed.2021.105137 34953358

[22] Wang, M. J. et al. Grey wolf optimization evolving kernel extreme learning machine: Application to bankruptcy prediction, *ENGINEERING APPLICATIONS OF ARTIFICIAL INTELLIGENCE*, vol. 63, pp. 54–68, AUG (2017).

[23] Yaqoob, A., Verma, N. K., Aziz, R. M. & Shah, M. A. RNA-Seq analysis for breast cancer detection: a study on paired tissue samples using hybrid optimization and deep learning techniques, *Journal of Cancer Research and Clinical Oncology*, vol. 150, no. 10, p. 455, /10/10 2024. (2024). 10.1007/s00432-024-05968-z PMC11467072 39390265

[24] Yaqoob, A., Verma, N. K., Aziz, R. M. & Shah, M. A. Optimizing cancer classification: a hybrid RDO-XGBoost approach for feature selection and predictive insights. *Cancer Immunol. Immunother.*, **73**, 12, p. 261, 2024/10/09 (2024). 10.1007/s00262-024-03843-x PMC11464649 39382649

[25] Afreen, S., Bhurjee, A. K. & Musheer Aziz, R. Cancer classification using RNA sequencing gene expression data based on Game Shapley local search embedded binary social ski-driver optimization algorithms, *Microchemical Journal*, vol. 205, p. 111280, /10/01/ 2024. (2024).

[26] Yaqoob, A., Verma, N. K. & Aziz, R. M. Improving breast cancer classification with mRMR + SS0 + WSVM: a hybrid approach. *Multimedia Tools Appl.*, 2024/09/06 (2024).

[27] Aziz, R. M., Hussain, A. & Sharma, P. Cognizable crime rate prediction and analysis under Indian penal code using deep learning with novel optimization approach. *Multimedia Tools Appl.*, **83**, 8, pp. 22663–22700, 2024/03/01 (2024).

[28] Joshi, A. A. & Aziz, R. M. Soft Computing techniques for Cancer classification of gene expression microarray data: A Three-Phase Hybrid Approach, in Computational Intelligence for Data Analysis: Bentham Science, 92–113. (2024).

[29] Li, J. & Li, M. Sep, Prediction of ultra-short-term wind power based on BBO-KELM method. *J. Renew. Sustain. Energy*, **11**, 5, (2019). Art. 056104.

[30] Quan, R., Liang, W. L., Wang, J. H., Li, X. R. & Chang, Y. F. An enhanced fault diagnosis method for fuel cell system using a kernel extreme learning machine optimized with improved sparrow search algorithm. *Int. J. Hydrog. Energy*. **50**, 1184–1196 (2024).

[31] Dai, Y. et al. Modeling of thermal error electric spindle based on KELM ameliorated by snake optimization. *Case Stud. Therm. Eng.*, **40**, (2022), Art. 102504.

[32] Han, X. S., Shi, Y., Tong, R. J., Wang, S. T. & Zhang, Y. Research on short-term load forecasting of power system based on IWOA-KELM. *Energy Rep.* **9**, 238–246 (2023).

[33] Yue, Y. G., Cao, L., Chen, H. S., Chen, Y. D. & Su, Z. G. Towards an Optimal KELM Using the PSO-BOA Optimization Strategy with Applications in Data Classification, *Biomimetics*, vol. 8, no. 3, Jul Art. no. 306. (2023). 10.3390/biomimetics8030306 PMC10807650 37504194

[34] Arora, S. & Singh, S. J. S. Butterfly optimization algorithm: a novel approach for global optimization, vol. 23, pp. 715–734, (2019).

[35] Wolpert, D. H. & Macready, W. G. No free lunch theorems for optimization. *IEEE Trans. Evol. Comput.* **1** (1), 67–82 (1997).

[36] Abdel-Basset, M., Abdel-Fatah, L. & Sangaiah, A. K. Chapter 10 - Metaheuristic algorithms: a Comprehensive Review, in Computational Intelligence for Multimedia Big Data on the Cloud with Engineering Applications, (eds Sangaiah, A. K., Sheng, M. & Zhang, Z.) Academic, 185–231. (2018).

[37] El-kenawy, E. S. M. et al. Greylag Goose Optimization: Nature-inspired optimization algorithm, *Expert Systems with Applications*, vol. 238, p. 122147, /03/15/ 2024. (2024).

[38] Zamani, H., Nadimi-Shahraki, M. H. & Gandomi, A. H. QANA: Quantum-based avian navigation optimizer algorithm. *Eng. Appl. Artif. Intell.* **104**, 104314 (2021). 2021/09/01/.

[39] Mehta, P., Kumar, S., Tejani, G. G., khishe, M. & MOBBO. : A Multiobjective Brown Bear Optimization Algorithm for Solving Constrained Structural Optimization Problems, **2024**(1), p. 5546940 (2024).

[40] Tejani, G. G., Mashru, N., Patel, P., Sharma, S. K. & Celik, E. Application of the 2-archive multi-objective cuckoo search algorithm for structure optimization. *Sci. Rep.*, **14**, 1, p. 31553, 2024/12/30 2024. 10.1038/s41598-024-82918-2 PMC11685762 39738304

[41] Mirjalili, S. & Lewis, A. The Whale Optimization Algorithm, *ADVANCES IN ENGINEERING SOFTWARE*, Article vol. 95, pp. 51–67, 2016 MAY 2016.

[42] El-kenawy, E. M. & A. F. J., A. R. J. o. A. I. Ibrahim, and Metaheuristics, Football Optimization Algorithm (FbOA): A Novel Metaheuristic Inspired by Team Strategy Dynamics, (2024).

[43] Faramarzi, A., Heidarinejad, M., Mirjalili, S. & Gandomi, A. H. Marine predators Algorithm: a nature-inspired metaheuristic. *EXPERT Syst. Appl. Article* **152**, (2020), Art. 113377.

[44] Fu, Y., Liu, D., Chen, J. & He, L. Secretary bird optimization algorithm: a new metaheuristic for solving global optimization problems. *Artif. Intell. Rev.*, **57**, 5, p. 123, 2024/04/23 (2024).

[45] Chandra, B. et al. Fuzzy SLIQ decision tree algorithm, vol. 38, no. 5, pp. 1294–1301, (2008). 10.1109/TSMCB.2008.923529 18784012

[46] Chandra, B. & Paul Varghese, P. Fuzzifying Gini Index based decision trees. *Expert Syst. Appl.* **36** (4), 8549–8559 (2009). 2009/05/01/.

[47] Qiu, H., J. I. J. o., H., Zhang, M. E. & Science, C. Fuzzy SLIQ decision tree based on classification sensitivity, vol. 3, no. 5, p. 18, (2011).

[48] Deng, D. & Kasabov, N. J. N. On-line pattern analysis by evolving self-organizing maps, vol. 51, pp. 87–103, (2003).

[49] Šter, B. & Dobnikar, A. Neural networks in medical diagnosis: Comparison with other methods, in *International conference on engineering applications of neural networks*, pp. 427 – 30. (1996).

[50] Beloufa, F. & Chikh, M. A. Design of fuzzy classifier for diabetes disease using Modified Artificial Bee Colony algorithm, *Computer Methods and Programs in Biomedicine*, vol. 112, no. 1, pp. 92–103, /10/01/ 2013. (2013). 10.1016/j.cmpb.2013.07.009 23932385

[51] Mallika, C. & Selvamuthukumaran, S. A hybrid Crow Search and Grey Wolf Optimization Technique for Enhanced Medical Data Classification in diabetes diagnosis system. *Int. J. Comput. Intell. Syst.*, **14**, 1, p. 157, 2021/09/01 (2021).

[52] Hu, G., Du, B., Wang, X. & Wei, G. An enhanced black widow optimization algorithm for feature selection. *Knowl. Based Syst.* **235**, 107638 (2022).

[53] Hakli, H. & Kiran, M. S. An improved artificial bee colony algorithm for balancing local and global search behaviors in continuous optimization. *Int. J. Mach. Learn. Cybern*, **11**, 9, pp. 2051–2076, (2020).

[54] Yu, M. et al. Improved multi-strategy adaptive Grey Wolf optimization for practical engineering applications and high-dimensional problem solving. *Artif. Intell. Rev.*, **57**, 10, p. 277, 2024/09/05 (2024).

[55] Fu, Y., Liu, D., Fu, S. & Chen, J. and L. J. S. r. He, Enhanced aquila optimizer based on tent chaotic mapping and new rules, vol. 14, no. 1, p. 3013, (2024). 10.1038/s41598-024-53064-6 PMC11303773 38321037

[56] Huang, H. et al. Multi-strategy improved artificial rabbit optimization algorithm based on fusion centroid and elite guidance mechanisms, *Computer Methods in Applied Mechanics and Engineering*, vol. 425, p. 116915, /05/15/ 2024. (2024).

[57] Xie, J. et al. An enhanced snow ablation optimizer for UAV swarm path planning and engineering design problems, vol. 10, no. 18, (2024). 10.1016/j.heliyon.2024.e37819 PMC11417320 39315149

[58] Kuk-Hyun, H. & Jong-Hwan, K. Genetic quantum algorithm and its application to combinatorial optimization problem, in *Proceedings of the 2000 Congress on Evolutionary Computation. CEC00 (Cat. No.00TH8512)*, vol. 2, pp. 1354–1360 vol.2. (2000).

[59] Wu, R. et al. An improved sparrow search algorithm based on quantum computations and multi-strategy enhancement, vol. 215, no. C %J Expert Syst. Appl., p. 37, (2023).

[60] Zhu, F. et al. Dung beetle optimization algorithm based on quantum computing and multi-strategy fusion for solving engineering problems, *Expert Systems with Applications*, vol. 236, p. 121219, /02/01/ 2024. (2024).

[61] Sansawas, S. et al. Gaussian Quantum-Behaved Particle Swarm with Learning Automata-Adaptive Attractor and Local Search, in *19th International Conference on Electrical Engineering/Electronics, Computer, Telecommunications and Information Technology (ECTI-CON)*, 2022, pp. 1–4. (2022).

[62] Xiong, H., Wu, Z., Fan, H., Li, G. & Jiang, G. Quantum rotation gate in quantum-inspired evolutionary algorithm: A review, analysis and comparison study, *Swarm and Evolutionary Computation*, vol. 42, pp. 43–57, /10/01/ 2018. (2018).

[63] Kibria, B. G. & J., A. H. J. o. S. r. Joarder, A short review of multivariate t-distribution, vol. 40, no. 1, pp. 59–72, (2006).

[64] Xie, J. et al. An enhanced snow ablation optimizer for UAV swarm path planning and engineering design problems, *Heliyon*, vol. 10, no. 18, p. e37819, 2024/09/30/ (2024). 10.1016/j.heliyon.2024.e37819 PMC11417320 39315149

[65] Awad, N. H., Ali, M. Z. & Suganthan, P. N. Ensemble sinusoidal differential covariance matrix adaptation with Euclidean neighborhood for solving CEC2017 benchmark problems, in *2017 IEEE Congress on Evolutionary Computation (CEC)*, pp. 372–379. (2017).

[66] Kennedy, J. & Eberhart, R. Particle swarm optimization, in *Proceedings of ICNN’95 - International Conference on Neural Networks*, vol. 4, pp. 1942–1948 vol.4. (1995).

[67] Mirjalili, S., Mirjalili, S. M. & Lewis, A. Grey Wolf Optimizer. *Adv. Eng. Softw. Article* **69**, pp. 46–61 (2014).

[68] Xue, J. & Shen, B. Dung beetle optimizer: a new meta-heuristic algorithm for global optimization, *Journal of Supercomputing*, Article; Early Access (2022).

[69] Chopra, N. & Mohsin Ansari, M. Golden jackal optimization: A novel nature-inspired optimizer for engineering applications, *Expert Systems with Applications*, vol. 198, p. 116924, /07/15/ 2022. (2022).

[70] Wang, J., Wang, W., Hu, X., Qiu, L. & Zang, H. J. A. I. R. Black-winged kite algorithm: a nature-inspired meta-heuristic for solving benchmark functions and engineering problems, vol. 57, no. 4, pp. 1–53, (2024).

[71] Abdel-Basset, M., Mohamed, R. & Abouhawwash, M. Crested Porcupine Optimizer: a new nature-inspired metaheuristic. *Knowl. Based Syst.* **284**, 111257 (2024). 2024/01/25/.

[72] Mohanty, F., Rup, S., Dash, B., Majhi, B. & Swamy, M. J. A. S. C. An improved scheme for digital mammogram classification using weighted chaotic salp swarm algorithm-based kernel extreme learning machine, **91**, p. 106266, (2020).

[73] Zhou, Z., Chen, J. & Zhu, Z. J. N. Regularization incremental extreme learning machine with random reduced kernel for regression, vol. 321, pp. 72–81, (2018).

[74] Wang, M. et al. Toward an optimal kernel extreme learning machine using a chaotic moth-flame optimization strategy with applications in medical diagnoses, vol. 267, pp. 69–84, (2017).

[75] Lv, L., Wang, W. & Zhang, Z. and X. J. K.-b. s. Liu, a novel intrusion detection system based on an optimal hybrid kernel extreme learning machine, **195**, p. 105648, (2020).

[76] Li, Y. et al. Epileptic seizure detection in EEG signals using sparse multiscale radial basis function networks and the Fisher vector approach, **164**, pp. 96–106, (2019).

[77] Zhang, X. et al. Pyramid channel-based feature attention network for image dehazing, **197**, p. 103003, (2020).

[78] Li, Q. et al. An Enhanced Grey Wolf Optimization Based Feature Selection Wrapped Kernel Extreme Learning Machine for Medical Diagnosis, vol. p. 9512741:1-9512741:15, 2017. (2017). 10.1155/2017/9512741 PMC5299219 28246543

